# Ultrafast Spectroscopy Reveals Significant Differences in LH2 Exciton Mobility at Cryogenic and Ambient Temperatures

**DOI:** 10.1021/acs.jpclett.5c03917

**Published:** 2026-02-13

**Authors:** Erika Keil, Pavel Malý, Richard J. Cogdell, Jürgen Hauer, Donatas Zigmantas, Erling Thyrhaug

**Affiliations:** † 9184 Technical University of Munich, School of Natural Sciences, Department of Chemistry, Lichtenbergstrasse 4, 85748 Garching, Germany; ‡ Faculty of Mathematics and Physics, 138735 Charles University, Prague, 11336, Czech Republic; § School of Molecular Biosciences, 3526 University of Glasgow, Room 402 Davidson Building, Glasgow G12 8QQ, Scotland; ∥ Chemical Physics, 5193 Lund University, Naturvetarvägen 16, 22362 Lund, Sweden

## Abstract

Spectroscopic studies of energy transport through the photosynthetic apparatus have been crucial to expanding our understanding of biological energy conversion. Correlating spectroscopic information to the electronic structure and function in these complex systems remains highly challenging, however. While cryogenic experimental conditions help in improving the effective spectral resolution and sample stability, the observed fine-grained dynamics do not necessarily reflect *in vivo* functionality. To address this issue, we target the temperature dependence of energy migration in light-harvesting complex 2 of purple bacteria. Temperature- and polarization-controlled two-dimensional electronic spectroscopy reveal rapid exciton immobilization at low temperatures, while intensity-dependent experiments allow identification of transport barriers. We find that exciton trapping, dominating the dynamics at 80 K, becomes negligible above 150 K, implying that observations at cryogenic temperatures do not always directly reflect biological function. We additionally find that considerable care and explicit modeling may be necessary for correct interpretation of multiexciton experiments.

## Graphical abstract

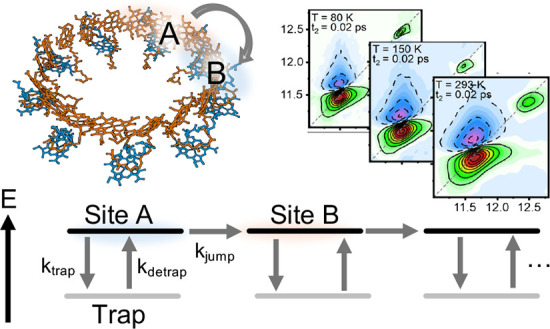

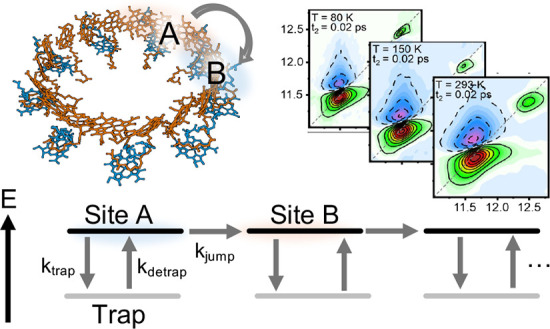

The peripheral Light-Harvesting Complex 2 (LH2) is a ringlike pigment–protein complex (PPC) found in purple phototrophic bacteria, where its main role is to increase the effective absorption cross-section per photosynthetic reaction center. Its global structure is formed from the assembly of 7 to 9 apoprotein dimers depending on bacterial species—the so-called α/β subunits.^1–4^ In most LH2 variants, each of these subunits contains two polypeptides that bind a moderately strongly coupled pair of bacteriochlorophyll a (BChl) pigments, a weakly coupled BChl, and one of several possible carotenoids (Supporting Information, Figure S1), although exceptions to this motif are known.^3,4^ The arrangement of BChl pigments within the subunits results in the formation of two ring-like pigment structures in the assembled PPC, each of which displays near-Gaussian band-like optical absorption features in the near-infrared region (Supporting Information, Figure S1). These absorption bands and the associated ring structures are generally referred to as the B800 and B850 band/ring, respectively.^1,5^

The functionality of LH2 is based on light capture by these pigment rings, followed by a complex, ultrafast sequence of intra- and inter- ring transport processes. In the complete photosynthetic unit, the excitation energy is ultimately transferred from LH2 to the core antenna Light-Harvesting complex 1 (LH1),^6–8^ where the photosynthetic reaction center is hosted. This energy transfer network based on structures of almost isoenergetic pigments features highly congested spectra in which resolvable detail is lost due to static disorder and thermal broadening.

To alleviate the reoccurring problem of spectral congestion in a wide range of PPCs, it is common practice to probe the energy transfer dynamics in these at cryogenic temperatures. Under these conditions, thermal broadening in the system decreases, leading to sharper spectral lines and a decongestion of spectral signatures.^9^ As such, experiments at cryogenic temperatures greatly facilitate the extraction of accurate photoinduced dynamics. Cryogenic conditions are clearly different than the native (physiological) environment of PPCs, however, which may lead to non-negligible differences in the photophysics of the system, since thermal fluctuations can change transition energies and excitonic couplings—and therefore the overall energy landscape.^10^ In LH2, for example, the line shape and energy gap between B800 and B850 bands change significantly with temperature^11^ (Supporting Information, Figure S1), an effect which has been attributed to the lowering of nearest-neighbor excitonic couplings.^12,13^ Additionally, energy transfer dynamics typically slow down moderately in conjunction with lowering of the temperature^14^ (Supporting Information, Figure S2).

In most cases, regardless of other experimental conditions, the ultrafast spectroscopy experiments used to characterize energy transfer pathways in PPCs rely on inducing purely single-excitation (single-particle) dynamics. Therefore, multiparticle processes such as exciton–exciton annihilation (EEA) are usually unwanted contributions. It has been shown, however, that multiparticle processes can provide valuable insight into properties such as exciton transport.^15–18^ In particular, the reliable extraction of multiparticle dynamics based on controlled experimental conditions enables the determination of functional properties that define spatial transport.

In this work, our goal is to bridge the gap between experiments targeting the functional description of light-harvesting at cryogenic and ambient temperatures. To achieve this, we rely on polarization- and temperature-controlled two-dimensional electronic spectroscopy (2DES) and transient grating (TG) spectroscopy. As a model system, we focus on exciton motion in LH2 extracted from *Rhodopseudomonas acidophila* at a range of temperatures. In agreement with earlier work,^19,20^ we observe exciton trapping at low temperatures, and we use ultrafast spectroscopy to estimate the trapping rates and trap depths. Using polarization-controlled 2DES, we follow the exciton mobility by tracking the signal depolarization under different excitation and detection conditions. As a direct marker of exciton mobility, we further follow exciton–exciton annihilation in the system by decomposing a series of power-dependent TG measurements into nonlinear response contributions. Finally, we compare the experimental data with a simplified numerical model to explain the observations.

We present absorptive 2DES spectra of LH2 from *Rps. acidophila* under low-energy (annihilation-free) excitation conditions (pulse energy: 0.6 nJ) at selected temperatures and population times *t*_2_ in Figure 1. The early time 2DES spectra show two positive diagonal features corresponding to the B800 and B850 absorption bands. B800 appears as an isolated sharp feature, while the lower-energy B850 band is broader with a strongly distorted line shape due to overlap with a negative-amplitude excited-state absorption (ESA) feature at the high-energy side. In agreement with earlier work,^2,7,21,22^ we find that, besides the ground-state recovery at long times, energy transfer occurs on three main time scales: (i) sub-100 fs loss of signal amplitude and excitation/detection frequency correlation; (ii) energy relaxation toward the bottom of both bands over a few hundred fs; and (iii) B800→B850 transfer, recognizable by the appearance of a distinctive below-diagonal cross-peak, on a ps time scale. In general, we find that the rates of the latter two processes increase at higher temperatures (Supporting Information, Figure S2).

**Figure 1:**
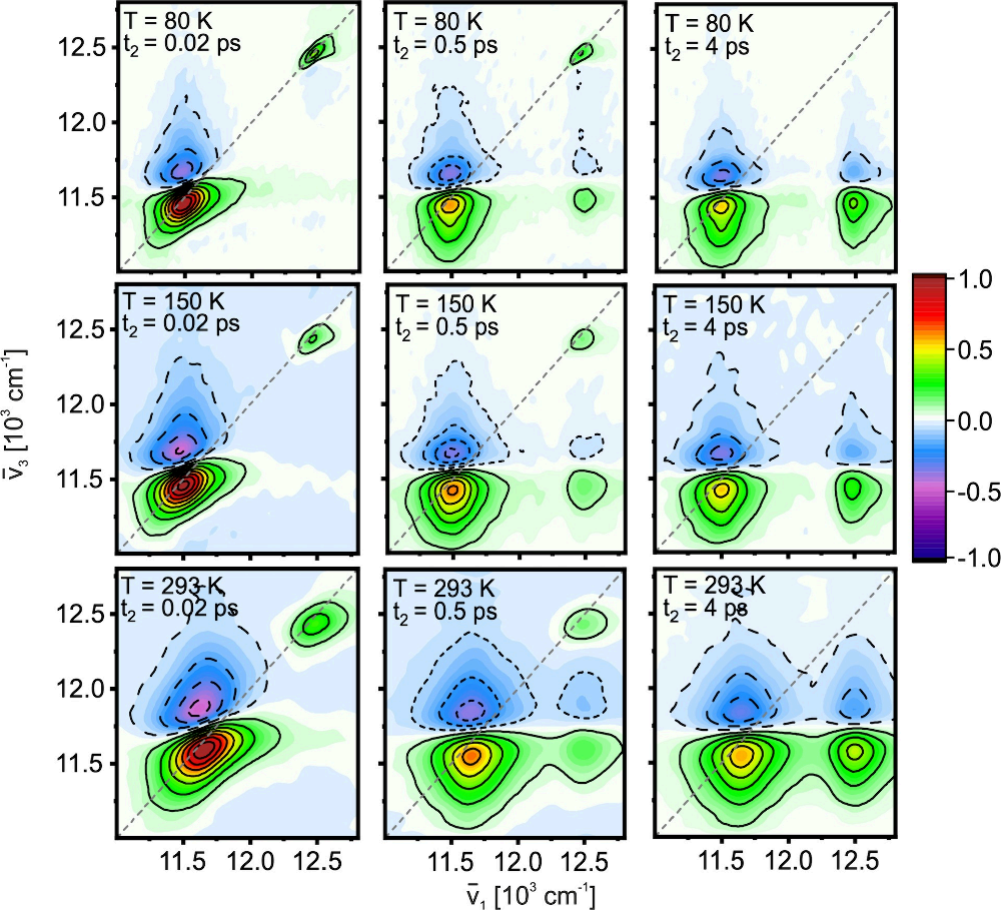
Absorptive 2DES spectra of LH2 recorded at magic angle polarization conditions at selected population times (columns) and temperatures (rows). Here, *ν̅*_1_ is the frequency of excitation, while *ν̅*_3_ is the detection frequency. The spectra are normalized to the amplitude maximum of the spectra at 20 fs for each temperature. Note the broadening of the line widths at increasing temperature.

In this work, we focus on dynamics within the primary light-harvesting structure of LH2: the moderately strongly coupled B850 band. The corresponding analysis of the weakly coupled B800 band can be found in the Supporting Information (Figures S3, S4).

The B850 intraband dynamics have been investigated by 2DES in earlier work.^19,20,23^ However, single-excitation experiments at individual temperatures provide limited information about the energy landscape and transport in complex systems such as LH2. Thus, we expand on the transport properties of LH2 by providing a comprehensive analysis of how exciton motion and relaxation dynamics are affected by the temperature. We quantitatively investigate the complex LH2 intraband dynamics by “slicing” the 2DES data sets along *ν̅*_1_ to generate a set of transient-absorption-like data at a given excitation frequency. Global kinetic analysis (GA) on the resulting data, summarized in Figure 2, allows for a detailed correlation of excitation energy and relaxation dynamics.^24^

**Figure 2:**
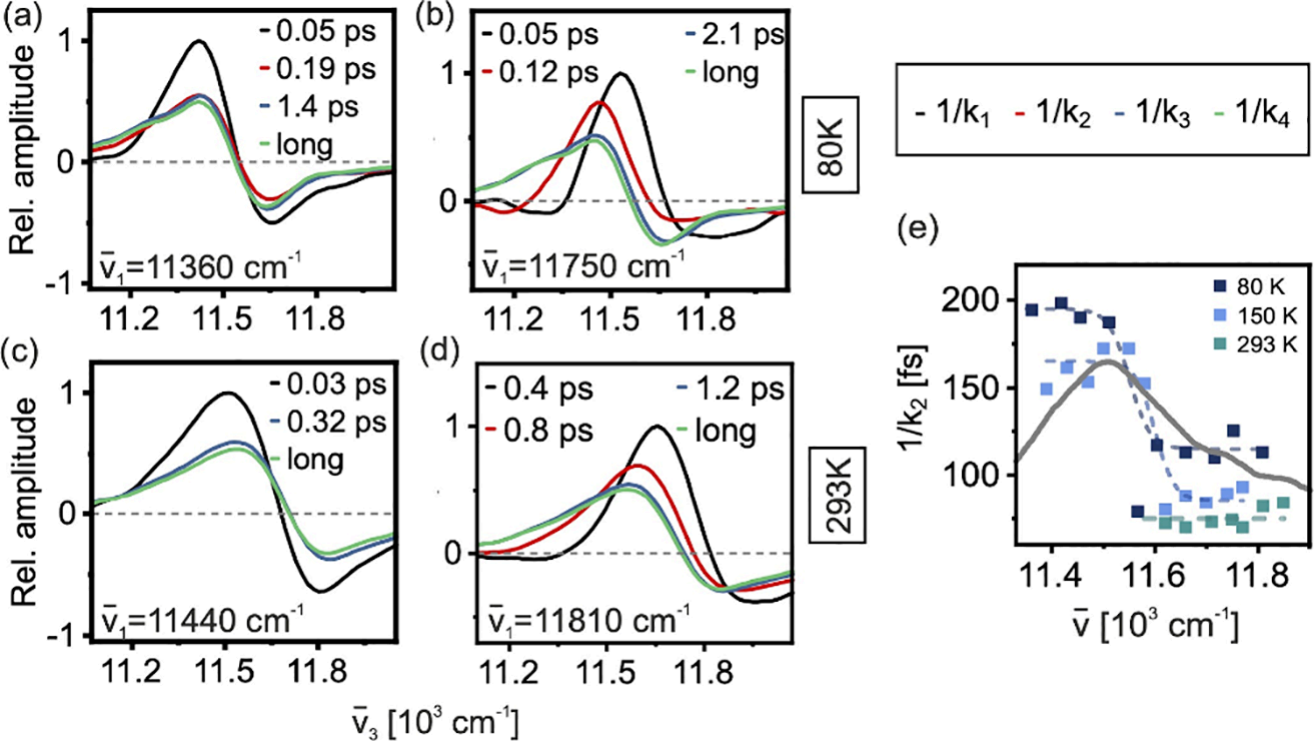
Kinetic analysis of the dynamics within the B850 band at a range of temperatures. Global kinetic spectra (EAS) at different temperatures (80 K: panels a and b, 293 K: panels c and d) and different excitation wavenumbers (red-edge excitation: panels a and c, blue-edge excitation: panels b and d). Panel e: intraband relaxation time-constant (1/*k*_2_) as a function of temperature with sigmoidal fits to guide the eye. See the text for details.

Immediately after excitation, we observe essentially temperature- and excitation frequency- independent spectral changes taking place on a time scale of ∼ 50 fs (*k*_1_). This ultrafast band-broadening in conjunction with loss of overall signal amplitude (EAS as black lines, Figure 2a-d) is consistent with observations in experimental^19,20,25^ and theoretical^26–28^ work, and it has been interpreted as dephasing of the initially excited wavepacket to form localized excitons. We note that this time-constant is close to the width of the excitation-pulse cross-correlation; thus, extracting precise quantitative values is not reliable. The physics behind the decay characterized by the rate constant *k*_2_ will be discussed separately below. We further observe a minor component (*k*_3_) in the ps range, which appears unrelated to electronic energy relaxation (blue lines, Figure 2a-d). Due to its subtle effect on the spectra, we assign this to a small-scale structural relaxation. Finally, the signal decays with an excitation-wavelength-independent component (*k*_4_) of several hundred picoseconds, corresponding to recovery of the ground state (*k*_4_, green lines, Figure 2a-d).

The most striking dynamics occurs immediately following dynamic localization. We observe a component *k*_2_ (red lines, Figure 2a,b,d) associated with intraband energy relaxation which is strongly excitation-frequency dependent in both lifetime and spectral shape, with faster relaxation after blue edge excitation. The line shape of the EAS at blue-edge excitation, characterized by a redshift from *k*_2_ to *k*_3_ (red lines, Figure 2b,d), as well as the dispersive shape of the corresponding DAS (Supporting Information, Figure S5) indicate that this component is related to downhill energy transfer. After red-edge excitation, on the other hand, the *k*_2_ EAS (red line, Figure 2a) is almost identical to that of the fully relaxed long-time spectrum (*k*_4_, green line, Figure 2a). This demonstrates that the lowest-energy exciton can be excited directly, implying a spatial extent substantially smaller than the optically dark k = 0 Bloch-wave expected in the absence of disorder.^29,30^

In earlier work performed at 80 K, we analyzed the intraband relaxation in detail^19,20^ and correlated energy relaxation to spatial exciton motion via comparison of spectral dynamics and the depolarization rate of the emitted signal field—the anisotropy decay. This correlation can be made, as spatial motion of excitons around the ring-like LH2 is necessarily connected to a change in transition dipole direction. As such, the anisotropy decay rate provides an estimate of the ensemble-averaged exciton transport velocity. While we have observed anisotropy decay rates consistent with earlier work^27,31,32^ through much of the B850 band, after excitation toward the red spectral edge we observed persistent high anisotropy—implying a lack of spatial motion. Nevertheless, we could identify fast energy-relaxation (here denoted as the kinetic component *k*_2_). This phenomenon has elsewhere been interpreted as exciton self-trapping or polaron formation.^33–35^

Unlike the other ultrafast processes within B850, *k*_2_ is strongly temperature-dependent, getting faster with increasing temperature (Figure 2e). At temperatures above 150 K, *k*_2_ is no longer needed to obtain a good fit of the data at the red edge (Figure 2c,e), suggesting that trapping does not significantly contribute to the dynamics at and above this temperature. Temperature-dependent transient anisotropy experiments yield a similar result: at low temperatures, a lack of depolarization after red-edge -excitation and -detection suggests low exciton mobility and thus trapping. However, at 150 K and above, we observe fast (<100 fs) depolarization to the anisotropy value of r = 0.1 instead (Supporting Information, Figure S6), indicating highly mobile excitons.

Overall, our observations suggest a shallow trap at the red edge of the B850 band that only significantly influences the dynamics below 150 K. Above this, the thermal energy is apparently sufficient to efficiently overcome the trap barrier, regardless of initial excitation energy. The depth of the trap state can thus be estimated to be of the order of *k*_B_ *T*(150 K) ≈ 104 cm^–1^.

While a kinetic analysis of the 2DES data combined with the anisotropy results gives insights into the energies and spectra of the initial and final states that participate in energy relaxation, they contain limited information about transport further along the antenna ring. In particular: while anisotropy reports on spatial exciton motion, depolarization is rapid, and the observable time scale is limited to a few next-neighbor jumps.

An alternative reporter on spatial transfer dynamics is based on monitoring multi -particle or -quasiparticle interactions, here exemplified by exciton–exciton annihilation (EEA).^36,37^ Signals from multiparticle interactions such as EEA are frequently mixed into the single-particle signals obtained via techniques such as Transient Absorption (TA) spectroscopy and 2DES. Normally, EEA is not a desirable contribution, as it distorts the single-particle dynamics.^38^ However, being a two-particle process, the EEA reports directly on exciton mobility.^17,18,39^

Malý and co-workers recently developed an approach to separate single- from multiparticle processes in nonlinear spectroscopy experiments.^15,40^ This approach relies on *n*-particle signals manifesting as an *n*-th order nonlinearity in the intensity dependence of the total signal. For TA with a weak probe (single interaction with the probe pulse), the total pump-intensity-dependent TA signal *PP*(*ω,T,I*) can be decomposed into^15^

1 $$\begin{array}{c} PP\left(\omega ,T,I\right)=PP^{\left(3\right)}\left(\omega ,T\right)I+PP^{\left(5\right)}\left(\omega ,T\right)I^{2}+... \end{array}$$

where PP^(2n+1)^ (*ω,T*) is the n-th order nonlinear response term, reflecting dynamics of up to *n* particles, and *I* is the intensity of the interacting fields. Recently, the approach has been formulated in a general form valid for 2DES as well.^41^

Here, we expand the approach to intensity-dependent transient grating (TG) experiments. In our TG experiments, the intensity of all four pulses (three plus local oscillator) is varied simultaneously, and the intensity dependence of the signal can be written as

2 $$\begin{array}{c} TG\left(\omega ,T,I\right)={TG}^{\left(3\right)}\left(\omega ,T\right)I^{2}+{TG}^{\left(5\right)}\left(\omega ,T\right)I^{3}+... \end{array}$$

As seen by comparison of Eqns. [Disp-formula eq1] and ([Disp-formula eq2]), for TG already the third-order signal is proportional to *I* ^2^. As in ref 15, we perform *N* intensity-dependent TG experiments to generate a set of *N* linear equations for the first *N* nonlinear order terms. Specifically, for *N* = 3 and intensities *I* = {α_1_, α_2_, α_3_}*I*_0_, we have

$$\left(\begin{array}{c} TG\left(\alpha_{1}I_{0}\right)\\ TG\left(\alpha_{2}I_{0}\right)\\ TG\left(\alpha_{3}I_{0}\right) \end{array}\right)=\left(\begin{array}{ccc} \alpha_{1}^{2} & \alpha_{1}^{3} & \alpha_{1}^{4}\\ \alpha_{2}^{2} & \alpha_{2}^{3} & \alpha_{2}^{4}\\ \alpha_{3}^{2} & \alpha_{3}^{3} & \alpha_{3}^{4} \end{array}\right)\left(\begin{array}{c} TG^{\left(3\right)}I_{0}^{2}\\ TG^{\left(5\right)}I_{0}^{3}\\ TG^{\left(7\right)}I_{0}^{4} \end{array}\right)$$

Inverting the relational matrix, we obtain

$$\left(\begin{array}{c} TG^{\left(3\right)}I_{0}^{2}\\ TG^{\left(5\right)}I_{0}^{3}\\ TG^{\left(7\right)}I_{0}^{4} \end{array}\right)={\left(\begin{array}{ccc} \alpha_{1}^{2} & \alpha_{1}^{3} & \alpha_{1}^{4}\\ \alpha_{2}^{2} & \alpha_{2}^{3} & \alpha_{2}^{4}\\ \alpha_{3}^{2} & \alpha_{3}^{3} & \alpha_{3}^{4} \end{array}\right)}^{-1}\left(\begin{array}{c} TG\left(\alpha_{1}I_{0}\right)\\ TG\left(\alpha_{2}I_{0}\right)\\ TG\left(\alpha_{3}I_{0}\right) \end{array}\right)$$

For intensity ratios {α_1_, α_2_, α_3_} = {1,3,4}, we thus obtain three equations that can be used to decompose TG data into the signals of the contributing nonlinear orders:

$$TG^{\left(3\right)}{\left(T,\omega \right)I}_{0}^{2}=2TG\left(T,\omega ,I_{0}\right)-0.22TG\left(T,\omega ,3I_{0}\right)+0.063TG\left(T,\omega ,4I_{0}\right)$$

$$TG^{\left(5\right)}{\left(T,\omega \right)I}_{0}^{3}=-1.17TG\left(T,\omega ,I_{0}\right)+0.28TG\left(T,\omega ,3I_{0}\right)-0.08TG\left(T,\omega ,4I_{0}\right)$$

$$TG^{\left(7\right)}{\left(T,\omega \right)I}_{0}^{4}=0.17TG\left(T,\omega ,I_{0}\right)-0.06TG\left(T,\omega ,3I_{0}\right)+0.02TG\left(T,\omega ,4I_{0}\right)$$

In this work we use *I*_0_ = 0.5 nJ, 3*I*_0_ = 1.5 nJ, and 4*I*_0_ = 2 nJ in a series of experiments at the same temperatures for which 2DES was performed.

In each experiment, the intensity of the LO+signal after the sample was adjusted with a neutral density filter before detection to avoid saturation of the CCD camera while maximizing the signal-to-noise ratio. As a consequence, the data sets for different pulse intensities are not directly comparable and must be scaled to reflect the true signal amplitude at the chosen intensity ratios. The correct scaling can be ensured by exploiting the fact that the transient grating signal in the B800 region can be considered annihilation-free within the first couple of hundreds of fs (cf. Supporting Information, Figure S7 for representative kinetic traces in the B800 band at different pump powers) and thus reflects the single-excitation dynamics. We can therefore assume that the signal in this region scales with *I* ^2^ (cf. Eqn. [Disp-formula eq2]), calculate and thus verify the scaling factors to counter the attenuation of the LO+signal on the detector, and isolate the pure third- (*TG* ^(3)^(*T,ω*)), fifth- (*TG* ^(5)^(*T,ω*)), and seventh-order (*TG* ^(7)^(*T,ω*)) contributions according to the aforementioned procedure (Supporting Information, Figure S8). In the following, we limit our analysis to the third- and fifth-order signals due to the very small amplitude of the seventh-order signal.

The *TG* ^(3)^(*T,ω*) signal reflects the single exciton dynamics, as is also the case for the 2DES experiments assuming annihilation-free excitation conditions. Global kinetic analysis of these data requires three components to achieve a good fit (Supporting Information, Figure S9). The longest component τ_3_ (comparable to 1/*k*_3_ in the 2DES data, Figure 2(a) to (d)) represents ground state recovery, while the second longest component τ_2_ (comparable to 1/*k*_2_ in the 2DES data, Figure 2(a) to (d)) contains contributions from intraband relaxation processes as well as B800→B850 transfer. In the τ_2_ (1/*k*_2_) component, the strength of 2DES becomes apparent: as shown in Figure 2(a) to (d), 2DES allows for excitation frequency dependent discussion of relaxation dynamics, with clear differences in line shape for the *k*_2_-component after blue and red excitation. These aspects are lost in *TG* ^(3)^(*T,ω*), see Figure S8 (Supporting Information), as TG integrates over all excitation wavelengths. The fast time constant τ_1_ (<200 fs) which appears in the analysis is likely related to a combination of dephasing (expected at ∼ 50 fs) and intraband relaxation (expected at roughly a few hundred fs, see above). Overall, the third-order TG data is consistent with the 2DES results.

The fifth-order TG data, *TG* ^(5)^(*T,ω*), contain contributions from two-exciton dynamics (including EEA) in addition to single-exciton dynamics. In the B850 region, *TG* ^(5)^(*T,ω*) consists of two overlapping spectral features with lineshapes that correspond to ground state bleaching and stimulated emission (GSB/SE) and excited-state absorption (ESA) in the third-order data, albeit with opposite sign. The qualitatively identical lineshapes of the third- and fifth-order signal (beyond the change of sign) are expected, as these data report on the appearance of single excitons conditional on excitation of two independent excitons.^15^ We expect both the ground state recovery of single excitons and the B800→B850 transfer to appear in the TG^(5)^ experiment. In the analysis of the TG^(5)^ data, both of these components (τ_2_ and τ_3_, respectively) were fixed to the results obtained from the analysis of the 2DES and TG^(3)^ data.

In addition to the two fixed kinetic components, a third kinetic component on the order of hundreds of femtoseconds (τ_1_) is necessary to properly fit the fifth-order signal. It varies strongly with temperature (400 fs @ 80 K, 70 fs @ 293 K, Figure 3a,b, black line), as expected for EEA.^19,42^ We hence ascribe this component to EEA-dynamics. The lifetimes and EAS of all components are shown in Figure 3 and Figure S9 (Supporting Information).

**Figure 3:**
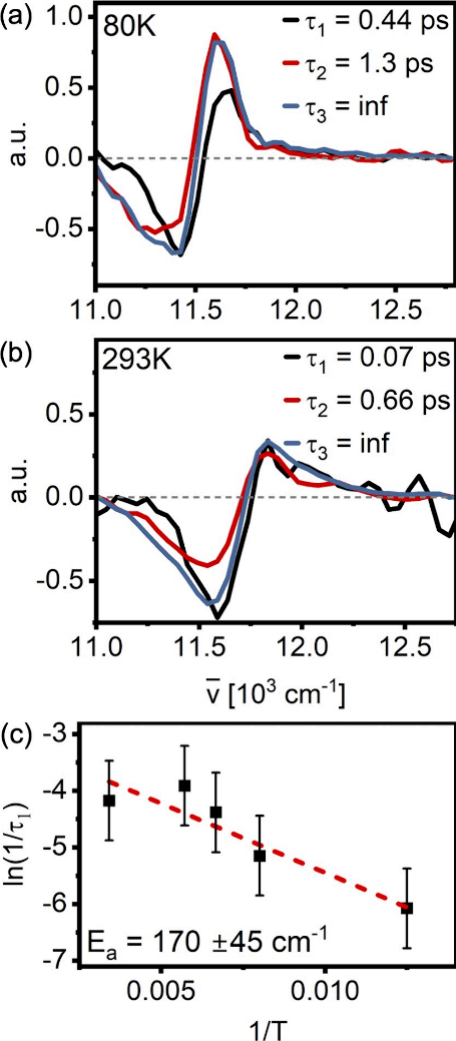
Evolution-associated spectra resulting from the global analysis of the fifth-order transient grating data sets at 80 K (panel a) and 293 K (panel b). The plots for the third-order data and the fifth order at 125, 150, and 175 K can be found in the Supporting Information (Figure S9). Panel c: temperature dependence of the EEA rate represented as an Arrhenius plot.

As EEA populates the doubly excited manifold with subsequent repopulation of the first excited state, we expect the signal associated with single excitons to rise with the EEA rate. At 80 K, we can see that this is clearly the case: In the ESA region, the total signal amplitude almost doubles after decay of the first component. In the GSB/SE region, the change is more subtle, likely due to an overlap with the ESA. However, the signal at the red edge of the B850 band also increases with the rate set by the first time constant, consistent with the interpretation that this component is associated with EEA. The rise of the red-edge GSB signal is also evident at higher temperatures, although a corresponding increase in the ESA amplitude is not distinguishable. This might be a consequence of broader lineshapes leading to a larger GSB/ESA overlap region.

In Figure 3c we show the temperature dependence of the rate constant *1/τ*_1_ of the EEA associated component. In an Arrhenius-type plot, the values of *ln­(1/τ*_1_ *)* lie in a straight line, suggesting that the observed temperature-dependence can be modeled as being due to a simple energy barrier. A linear fit of the *ln­(1/τ*_1_ *)* values results in an EEA ‘activation energy’ (*E*_a_) of ca. 170 cm^–1^ – considerably larger than the trap depth estimated from the temperature-dependent 2DES data. As such, it is evident that the effective barrier experienced by the system in a two-exciton process is larger than the corresponding barrier in the single-exciton case.

To qualitatively explain the discrepancy in trap depths extracted from 2DES and TG^(5)^ experiments, we implement a simple phenomenological transport model (shown schematically in Figure 4a). We take the B850 ring to consist of nine nonoverlapping effective sites. While it is generally understood that B850 excitons are delocalized over more than one site,^43^ thus resulting in partial spatial overlap of neighboring excitons, for simplicity we do not take this into account here. Exciton motion is taken to be a discrete random walk, where at each site, an exciton can either hop to a neighbor site with a rate *k*_*jump*_ or get trapped with a rate *k*_*trap*_ (Figure 4a). The hopping rate (*k*_*jump*_) is taken to be temperature independent. Once trapped, the detrapping rate is given by $k_{detrap}=$ ${k_{trap}\cdot e}^{-E_{trap}/k_{B}T}$ according to detailed balance. When two mobile excitons meet at the same site, they are annihilated. We do not, however, allow trapped excitons to annihilate. More details are given in the Supporting Information (Section 2).

**Figure 4:**
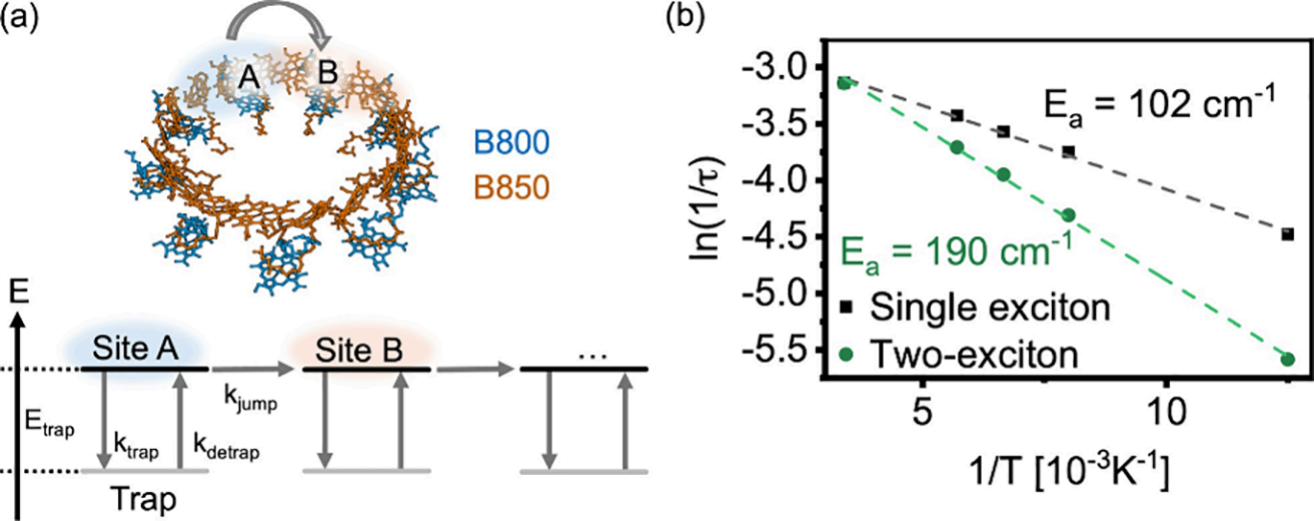
Panel a: schematic of the model used to simulate exciton transport in the B850 ring of LH2. The 18-Bchl ring is sectioned into nine sites. Excitons jump from site to site with a rate of *k*_*jump*_, which is identical for jumps between all effective sites. At each site, excitons can also get trapped with a rate *k*_*trap*_ into a trap with a depth *E*_*trap*_. The detrapping rate *k*_*detrap*_ is given by a detailed balance. Panel b: Activation energies retrieved from the Monte Carlo model for a mean lifetime τ of a single exciton assuming a fixed quencher site at the ring (black line) and for the mean lifetime τ of a pair of mobile excitons that undergo EEA when they meet (green line). In both cases, the excitons had a probability of getting trapped in a trap with a depth *E*_*trap*_ = 120 cm^–1^. See text for details.

We investigate the consistency of the model by randomly placing a single excitation in a ring containing one fixed quenching site and extracting the mean exciton lifetime τ. In line with expectations, without trapping, the dynamics are temperature-independent. If trapping is allowed (*E*_*trap*_ = 120 cm^–1^), the apparent activation energy (extracted from the Arrhenius equation fit) is slightly below the depth of the trap (Figure 4b, black line and dots). This accounts for the fact that some but not all excitons get trapped before reaching the quencher.

We then repeat the calculation for a scenario in which two excitons are placed on the ring, allowing EEA when they simultaneously arrive at the same site. In the absence of trapping, the resulting dynamics are again temperature independent (Supporting Information, Figure S10). When trapping is allowed (*E*_*trap*_ = 120 cm^–1^), however, the effective activation energy per exciton recovered from the temperature dependence of the mean annihilation time is larger than the trap depth (Figure 4b, green line and dots).

Overall, our simple phenomenological model shows that while the presence of traps clearly influences the dynamics, their effect on recovered single- and two-exciton dynamics is not identical. The apparent activation energy necessary to overcome (nonannihilating) traps generally appears larger in multiexciton experiments than what can be estimated from single-exciton measurements. We interpret this as being due to the larger probability of *any* trapping event happening in the (independent) two-exciton case, as both excitons have equal probability of being trapped before annihilation can take place. As a result, the mean number of barriers that must be overcome to yield an observable signal will be larger for a given transport distance in the two-exciton case compared to the single exciton case.

The parameters of our model point to some potential constraints on the traps in LH2: To achieve qualitative agreement between our model and the experimental findings, the EEA rate for the trapped excitons must be significantly smaller than that for the mobile ones. To rationalize this, we consider the microscopic mechanism of EEA. The initial step is energy transfer from a two-exciton state to a higher excited state. Analogously to single-exciton transfer, coupling between the respective transitions and energetic resonance is required. The conditions for transport thus being similar, it is plausible that trapping that renders excitons immobile also suppresses their annihilation. The details of our simple model can be found in the Supporting Information, section 2. A particular example of a trapping mechanism for which EEA can also be expected to be suppressed is exciton self-trapping, e.g., by a polaron formation, as suggested in previous work.^20,34,35^

We stress that our model is too simple to make definite claims about the physical nature of the trap states or the electronic structure of LH2. In particular: we here assume a fixed energy landscape with static traps that can be overcome by the additional thermal energy available at higher temperatures. However, our observations could also be explained by, e.g., dynamical changes in the complex structure, dynamical changes of the traps themselves, a weaker coupling to phonon modes at higher temperatures (if the trapping is indeed induced by polaron formation), or other nontrivial, temperature-dependent effects. It is also likely that the excitons themselves change with temperature (for instance, they may become smaller and more localized) and that this affects the trapping rate as well. Finally, in a strongly coupled system such as the B850 ring of LH2, even the factorization of the two-exciton states into exciton pairs is not exact, although it can be expected to be a good approximation.^44^ Within the framework of our experiments, we cannot distinguish between these possible effects. We do however believe that this work provides an interesting starting point and experimental constraints on observables for more refined calculations of exciton -transport and -interactions in this highly complex system, such as the ones presented in.^12^

In summary, we have investigated the temperature dependence of intracomplex energy transfer dynamics in LH2 by means of 2DES and TG spectroscopy. At cryogenic temperatures, the intraband dynamics in B850 are characterized by rapid immobilization of excitons at the low-energy side of the band. While this may seem counterintuitive in a PPC, our analysis of the 2DES data reveals that the traps are shallow (less than 120 cm^–1^) and their effect on mobility is negligible above ∼ 150 K.

Using polarization-controlled 2DES we can estimate an upper bound on the trap depth directly via the temperature dependence of the anisotropy decay. On the other hand, extracting the EEA kinetics from the fifth-order signal isolated from power-dependent TG measurements allows us to estimate a value for the average energy barrier that must be overcome during exciton motion within the band. Interestingly, the energy barrier that we extract from the fifth-order TG is substantially larger than the trap depth estimated from 2DES. In contrast to this observation, the underlying physics, the ‘real’ trap depth, clearly does not change depending on the experiment.

We rationalize this discrepancy by a comparison of single- and two-exciton dynamics in a simplified B850 model. In both cases we find that the experimentally recoverable ‘apparent trap depth’ deviates from the real value. This effect is due to the path for an exciton to travel a given distance involving, in general, not exactly one trapping event. Due to the possibility of being trapped multiple times, the effective energy barrier extracted from our model, both in the single- and two-exciton case, does not report directly on the trap depth but rather represents the energy barrier an exciton has to overcome on average to annihilate or reach the quenching site.

In summary, in this work we examine whether results from cryogenic ‘spectroscopic’ experimental conditions can be extrapolated to ambient ‘functional’ conditions for photosynthetic light-harvesting systems. We further demonstrate the advantages of analyzing and comparing the spectroscopic signatures of single- and multiparticle processes, which in sum reveal a more complete picture of the photophysical processes in light harvesting systems.

We stress that accurate interpretation of multiparticle experiments requires considerable care and, in general, likely explicit modeling. While the discrepancies observed in this work could be qualitatively understood by a simple transport model, we expect such comparison of parameters from single- and multiparticle experiments to be highly nontrivial as a rule.

While cryogenic experiments may be necessary to improve data quality, sample stability, or spectral resolution, our work highlights that results from these experiments do not trivially generalize to biological conditions. In the case of LH2, we find clear evidence for the extremely rapid immobilization of excitons at cryogenic temperatures. However, we find that these trapping dynamics in the B850 band do not affect the mobility at biologically relevant temperatures.

## Supplementary Material



## Data Availability

The data that support the findings of this study are publicly available for download on Zenodo (10.5281/zenodo.17670923) or can be obtained from the corresponding author upon reasonable request.

## Notes

Additional text and figures (SI.pdf) Details on experimental implementation and sample preparation (SI section 1). Additional temperature dependent UV/vis spectra and global kinetic spectra of the B800 region (Figures S1–S3). B800 anisotropy decays (Figure S4). Global kinetic spectra and anisotropy decays in the B850 region (Figures S5–S6). Intensity dependent dynamics in the B800 band (Figure S7). 3rd, 5th, and 7th order transient grating spectra, and global kinetic spectra of 3rd and 5th order TG experiments (Figures S8–S9). Details on transport model (SI section 2, Table T1 and Figure.S10) (PDF)

## References

[1] Blankenship, R. E. Molecular Mechanisms of Photosynthesis. 2nd ed.; Wiley/Blackwell: Chichester, West Sussex, 2012.

[2] Law C. J., Roszak A. W., Southall J., Gardiner A. T., Isaacs N. W., Cogdell R. J. (2004). The Structure and Function of Bacterial Light-Harvesting
Complexes. Mol. Membr. Biol..

[3] Gardiner A. T., Naydenova K., Castro-Hartmann P., Nguyen-Phan T. C., Russo C. J., Sader K., Hunter C. N., Cogdell R. J., Qian P. (2021). The 2.4 Å Cryo-EM
Structure of a Heptameric Light-Harvesting
2 Complex Reveals Two Carotenoid Energy Transfer Pathways. Sci. Adv..

[4] Qian P., Nguyen-Phan C. T., Gardiner A. T., Croll T. I., Roszak A. W., Southall J., Jackson P. J., Vasilev C., Castro-Hartmann P., Sader K. (2022). Cryo-EM structures of light-harvesting 2 complexes from *Rhodopseudomonas
palustris* reveal the molecular origin of absorption tuning. Proc. Natl. Acad. Sci. U.S.A..

[5] Cogdell R. J., Isaacs N. W., Freer A. A., Howard T. D., Gardiner A. T., Prince S. M., Papiz M. Z. (2003). The Structural Basis of Light-Harvesting
in Purple Bacteria. FEBS Lett..

[6] Saer R. G., Blankenship R. E. (2017). Light Harvesting
in Phototrophic Bacteria: Structure
and Function. Biochem. J..

[7] Mirkovic T., Ostroumov E. E., Anna J. M., Van Grondelle R., Govindjee, Scholes G. D. (2017). Light Absorption
and Energy Transfer in the Antenna Complexes of Photosynthetic Organisms. Chem. Rev..

[8] Cogdell R. J., Gall A., Köhler J. (2006). The Architecture
and Function of
the Light-Harvesting Apparatus of Purple Bacteria: From Single Molecules
to in Vivo Membranes. Q. Rev. Biophys..

[9] Zucchelli G., Garlaschi F. M., Jennings R. C. (1996). Thermal Broadening Analysis of the
Light Harvesting Complex II Absorption Spectrum. Biochemistry.

[10] Reiter S., Kiss F. L., Hauer J., de Vivie-Riedle R. (2023). Thermal Site
Energy Fluctuations in Photosystem I: New Insights from MD/QM/MM Calculations. Chem. Sci..

[11] Wu H. M., Reddy N. R. S., Cogdell R. J., Muenke C., Michel H., Small G. J. (1996). A Comparison of
the LH2 Antenna Complex of Three Purple
Bacteria by Hole Burning and Absorption Spectroscopies. Molecular Crystals and Liquid Crystals Science and Technology
Section A: Molecular Crystals and Liquid Crystals.

[12] Cupellini L., Jurinovich S., Campetella M., Caprasecca S., Guido C. A., Kelly S. M., Gardiner A. T., Cogdell R., Mennucci B. (2016). An *Ab Initio* Description of the Excitonic
Properties of LH2 and Their Temperature Dependence. J. Phys. Chem. B.

[13] Pajusalu M., Rätsep M., Trinkunas G., Freiberg A. (2011). Davydov Splitting of
Excitons in Cyclic Bacteriochlorophyll a Nanoaggregates of Bacterial
Light-Harvesting Complexes between 4.5 and 263 K. ChemPhysChem.

[14] Pullerits T., Hess S., Herek J. L., Sundström V. (1997). Temperature
Dependence of Excitation Transfer in LH2 of *Rhodobacter Sphaeroides*. J. Phys. Chem. B.

[15] Malý P., Lüttig J., Rose P. A., Turkin A., Lambert C., Krich J. J., Brixner T. (2023). Separating Single- from Multi-Particle
Dynamics in Nonlinear Spectroscopy. Nature.

[16] Malý P., Lüttig J., Turkin A., Dostál J., Lambert C., Brixner T. (2020). From Wavelike
to Sub-Diffusive Motion:
Exciton Dynamics and Interaction in Squaraine Copolymers of Varying
Length. Chem. Sci..

[17] Rehhagen C., Stolte M., Herbst S., Hecht M., Lochbrunner S., Würthner F., Fennel F. (2020). Exciton Migration in Multistranded
Perylene Bisimide J-Aggregates. J. Phys. Chem.
Lett..

[18] Stevens M. A., Silva C., Russell D. M., Friend R. H. (2001). Exciton
Dissociation
Mechanisms in the Polymeric Semiconductors Poly­(9,9-Dioctylfluorene)
and Poly­(9,9-Dioctylfluorene-Co-Benzothiadiazole). Phys. Rev. B Condens. Matter Mater. Phys..

[19] Keil E., Lokstein H., Cogdell R., Hauer J., Zigmantas D., Thyrhaug E. (2024). Light Harvesting in Purple Bacteria
Does Not Rely on
Resonance Fine-Tuning in Peripheral Antenna Complexes. Photosynth. Res..

[20] Thyrhaug E., Schröter M., Bukartė E., Kühn O., Cogdell R., Hauer J., Zigmantas D. (2021). Intraband
Dynamics and Exciton Trapping in the LH2 Complex of *Rhodopseudomonas
Acidophila*. J. Chem. Phys..

[21] Fleming G. R., Van Grondelle R. (1997). Femtosecond
Spectroscopy of Photosynthetic Light-Harvesting
Systems. Curr. Opin. Struct. Biol..

[22] Sundström V., Pullerits T., Van Grondelle R. (1999). Photosynthetic Light-Harvesting:
Reconciling Dynamics and Structure of Purple Bacterial LH2 Reveals
Function of Photosynthetic Unit. J. Phys. Chem.
B.

[23] Vulto S. I. E., Kennis J. T. M., Streltsov A. M., Amesz J., Aartsma T. J. (1999). Energy
Relaxation within the B850 Absorption Band of the Isolated Light-Harvesting
Complex LH2 from *Rhodopseudomonas Acidophila* at Low
Temperature. J. Phys. Chem. B.

[24] Van
Stokkum I. H. M., Larsen D. S., Van Grondelle R. (2004). Global and
Target Analysis of Time-Resolved Spectra. BBA
- Bioenergetics.

[25] Book L. D., Ostafin A. E., Ponomarenko I. N., Norris J. R., Scherer N. F. (2000). Exciton
Delocalization and Initial Dephasing Dynamics of Purple Bacterial
LH2. J. Phys. Chem. B.

[26] Chorošajev V., Rancova O., Abramavicius D. (2016). Polaronic
Effects at Finite Temperatures
in the B850 Ring of the LH2 Complex. Phys. Chem.
Chem. Phys..

[27] Sardjan A. S., Westerman F. P., Ogilvie J. P., Jansen T. L. C. (2020). Observation of
Ultrafast Coherence Transfer and Degenerate States with Polarization-Controlled
Two-Dimensional Electronic Spectroscopy. J.
Phys. Chem. B.

[28] Dahlbom M., Pullerits T., Mukamel S., Sundström V. (2001). Exciton Delocalization
in the B850 Light-Harvesting Complex: Comparison of Different Measures. J. Phys. Chem. B.

[29] Novoderezhkin V. I. (2025). Energy
Transfers between the B850 Antennas: Hybrid Hierarchical Equation
Approach. Photosynth. Res..

[30] Cupellini L., Caprasecca S., Guido C. A., Müh F., Renger T., Mennucci B. (2018). Coupling to
Charge Transfer States
Is the Key to Modulate the Optical Bands for Efficient Light Harvesting
in Purple Bacteria. J. Phys. Chem. Lett..

[31] Trinkunas G., Herek J. L., Polívka T., Sundström V., Pullerits T. (2001). Exciton Delocalization Probed by Excitation Annihilation
in the Light-Harvesting Atenna LH2. Phys. Rev.
Lett..

[32] Massey S. C., Ting P. C., Yeh S. H., Dahlberg P. D., Sohail S. H., Allodi M. A., Martin E. C., Kais S., Hunter C. N., Engel G. S. (2019). Orientational Dynamics of Transition
Dipoles and Exciton
Relaxation in LH2 from Ultrafast Two-Dimensional Anisotropy. J. Phys. Chem. Lett..

[33] Timpmann K., Katiliene Z., Woodbury N. W., Freiberg A. (2001). Exciton Self Trapping
in One-Dimensional Photosynthetic Antennas. J. Phys. Chem. B.

[34] Freiberg A., Rätsep M., Timpmann K., Trinkunas G., Woodbury N. W. (2003). Self-Trapped Excitons
in LH2 Antenna Complexes between
5 K and Ambient Temperature. J. Phys. Chem.
B.

[35] Polívka T., Pullerits T., Herek J. L., Sundström V. (2000). Exciton Relaxation
and Polaron Formation in LH2 at Low Temperature. J. Phys. Chem. B.

[36] Dostál J., Fennel F., Koch F., Herbst S., Würthner F., Brixner T. (2018). Direct Observation
of Exciton-Exciton Interactions. Nat. Commun..

[37] Valkunas L., Trinkunas G., Liuolia V., van Grondelle R. (1995). Nonlinear
Annihilation of Excitations in Photosynthetic Systems. Biophys. J..

[38] Brüggemann B., Christensson N., Pullerits T. (2009). Temperature Dependent Exciton-Exciton
Annihilation in the LH2 Antenna Complex. Chem.
Phys..

[39] Lin J. D. A., Mikhnenko O. V., Chen J., Masri Z., Ruseckas A., Mikhailovsky A., Raab R. P., Liu J., Blom P. W. M., Loi M. A. (2014). Systematic Study of Exciton Diffusion Length
in Organic Semiconductors by Six Experimental Methods. Mater. Horiz..

[40] Lüttig J., Mueller S., Malý P., Krich J. J., Brixner T. (2023). Higher-Order
Multidimensional and Pump-Probe Spectroscopies. J. Phys. Chem. Lett..

[41] Krich J. J., Brenneis L., Rose P. A., Mayershofer K., Büttner S., Lüttig J., Malý P., Brixner T. (2025). Separating Orders of Response in
Transient Absorption
and Coherent Multidimensional Spectroscopy by Intensity Variation. J. Phys. Chem. Lett..

[42] Brüggemann B., Christensson N., Pullerits T. (2009). Temperature Dependent Exciton-Exciton
Annihilation in the LH2 Antenna Complex. Chem.
Phys..

[43] Trinkunas G., Herek J. L., Polívka T., Sundström V., Pullerits T. (2001). Exciton Delocalization Probed by
Excitation Annihilation
in the Light-Harvesting Atenna LH2. Phys. Rev.
Lett..

[44] Abramavicius D., Voronine D. V., Mukamel S. (2008). Double-Quantum Resonances
and Exciton-Scattering
in Coherent 2D Spectroscopy of Photosynthetic Complexes. Proc. Natl. Acad. Sci. U.S.A..

