# Supplementary material for: Ultrafast Spectroscopy Reveals Significant Differences in LH2 Exciton Mobility at Cryogenic and Ambient Temperatures
Source: J Phys Chem Lett. 2026 Feb 13;17(8):2313–20. doi: 10.1021/acs.jpclett.5c03917 (PMC12951549; doi:10.1021/acs.jpclett.5c03917)
Supplement: Supplementary file 1 [file jz5c03917_si_001.pdf]

Supporting Information for:

Ultrafast spectroscopy reveals significant differences in LH2 exciton mobility at cryogenic and ambient temperatures

Erika Keil, Pavel Malý, Richard Cogdell, Jürgen Hauer, Donatas Zigmantas, and Erling Thyrhaug*

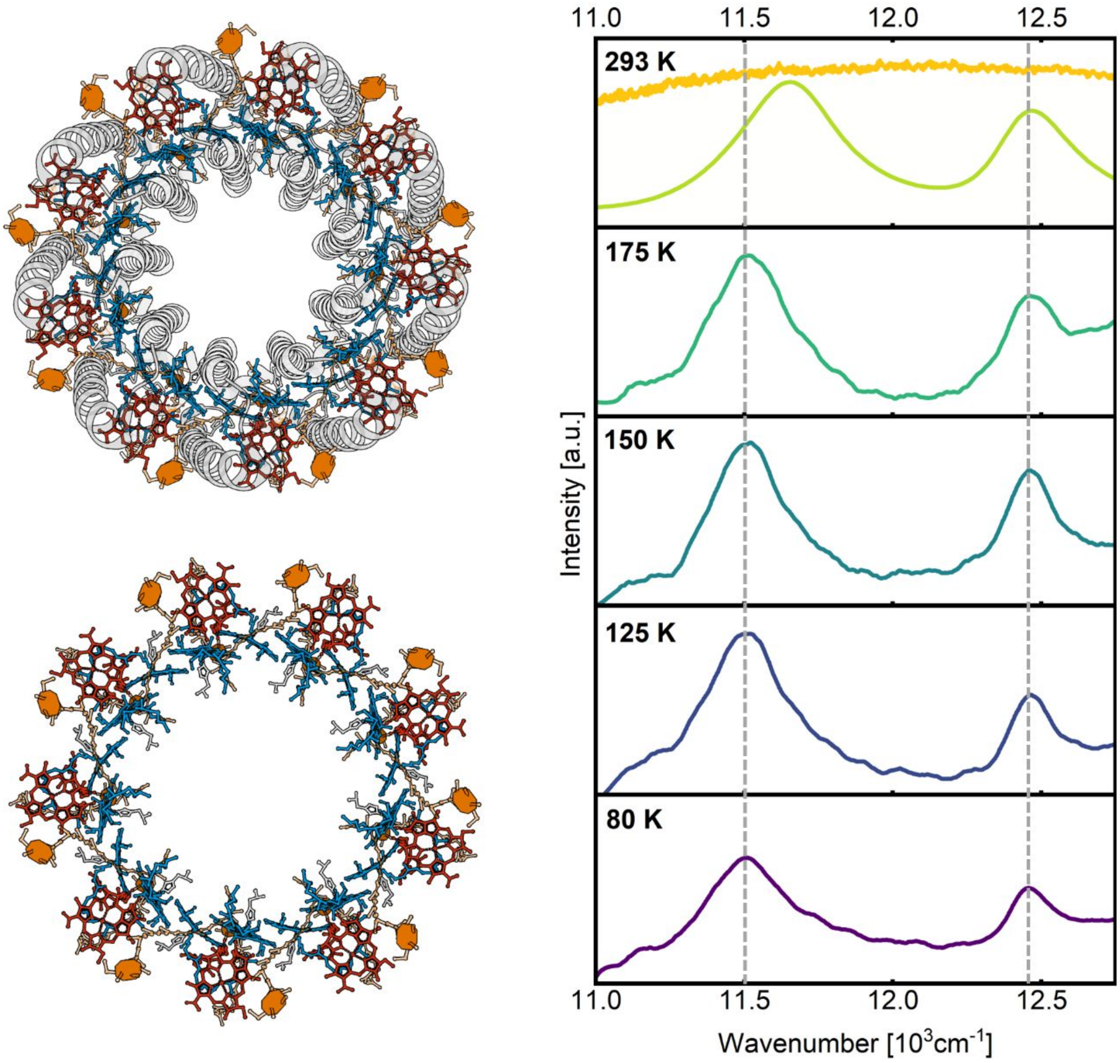

Figure S1

Left: Molecular structure of *Rps. acidophila* (PDB ID: 1NKZ). Protein chains are drawn in grey, carotenoids (rhodopin glucoside) in orange, B800 BChls in red and B850 BChls in blue.
Right: Absorption spectra of LH2 at 80 K, 125 K, 150 K, 175 K, and 293 K. The B800 band stays largely at the same position regardless of temperature, while the B850 band blue-shifts at higher temperatures, resulting in a narrowing of the B800-B850 gap. Absorption spectra below 293 K were retrieved from the local oscillator traces of the 2DES experiments. The panel at 293 K shows the NOPA spectrum used in the 2DES and TG experiments over the detection range.

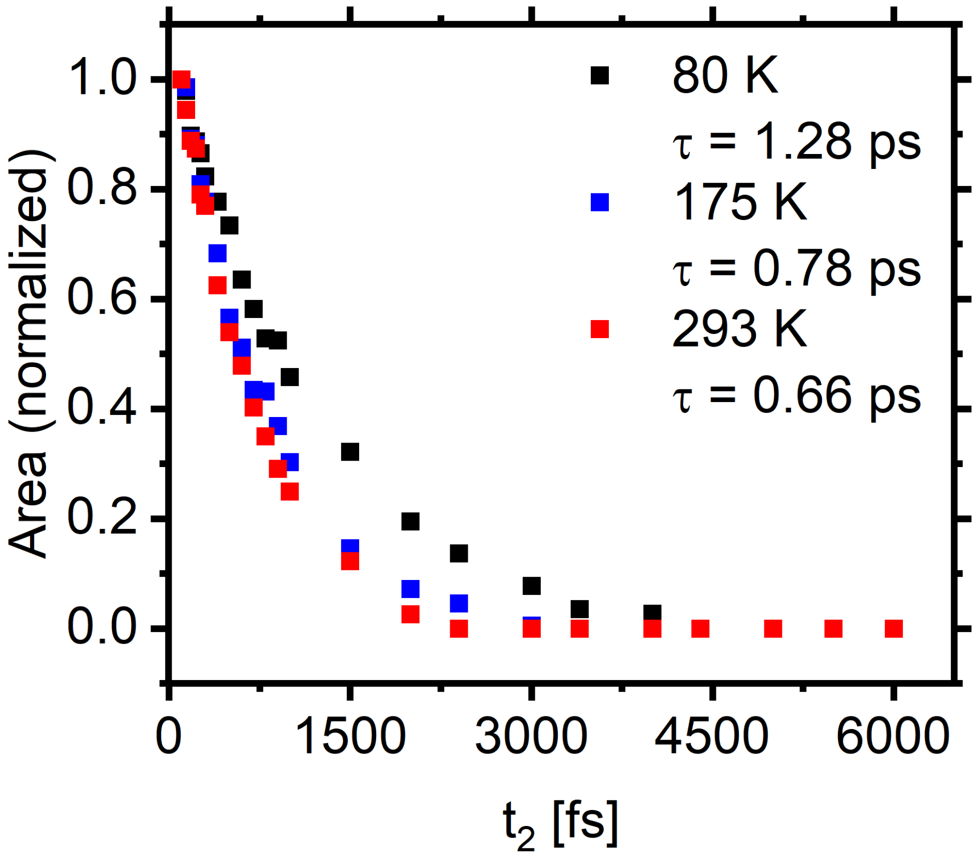

Figure S2

B800→B850 transfer rates at different temperatures. The rates were determined by integrating over the B800/B850 crosspeak area in the 2DES maps for each population time. The resulting decay was fitted by a monoexponential function to obtain the transfer rate.

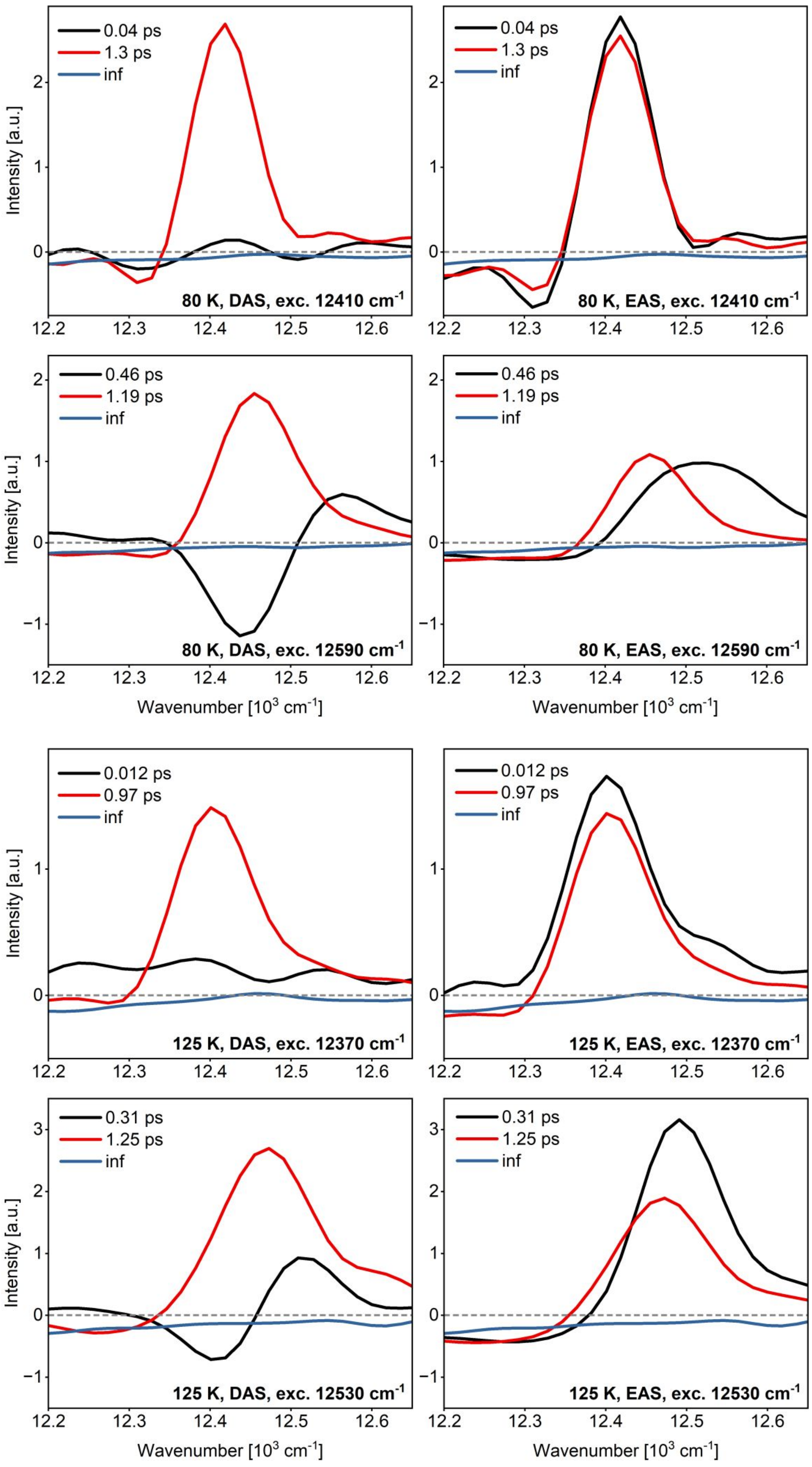

0.04 ps
1.3 ps
inf
80 K, DAS, exc. 12410 cm-1
80 K, EAS, exc. 12410 cm-1
0.46 ps
1.19 ps
inf
80 K, DAS, exc. 12590 cm-1
80 K, EAS, exc. 12590 cm-1
0.012 ps
0.97 ps
inf
125 K, DAS, exc. 12370 cm-1
125 K, EAS, exc. 12370 cm-1
0.31 ps
1.25 ps
inf
125 K, DAS, exc. 12530 cm-1
125 K, EAS, exc. 12530 cm-1
Intensity [a.u.]
Wavenumber [10^3 cm-1]

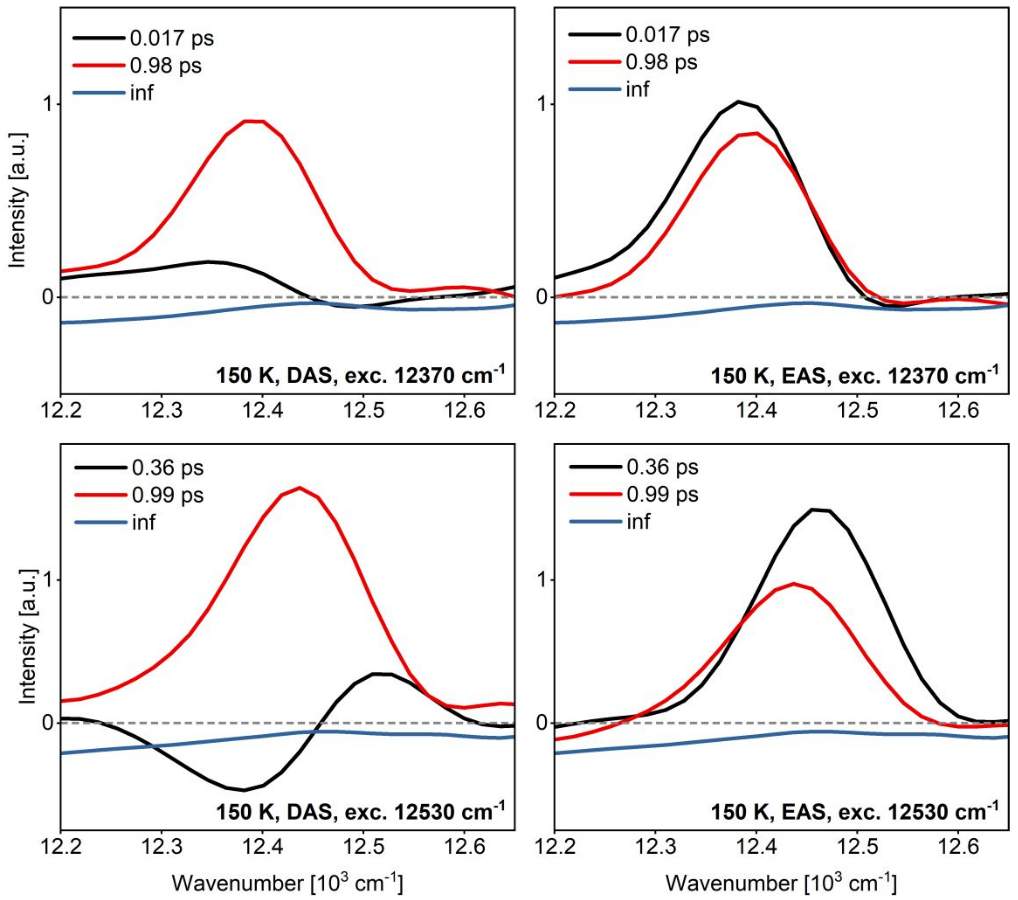
0.017 ps
0.98 ps
inf
150 K, DAS, exc. 12370 cm-1
150 K, EAS, exc. 12370 cm-1
0.36 ps
0.99 ps
inf
150 K, DAS, exc. 12530 cm-1
150 K, EAS, exc. 12530 cm-1
Intensity [a.u.]
Wavenumber [10³ cm-1]

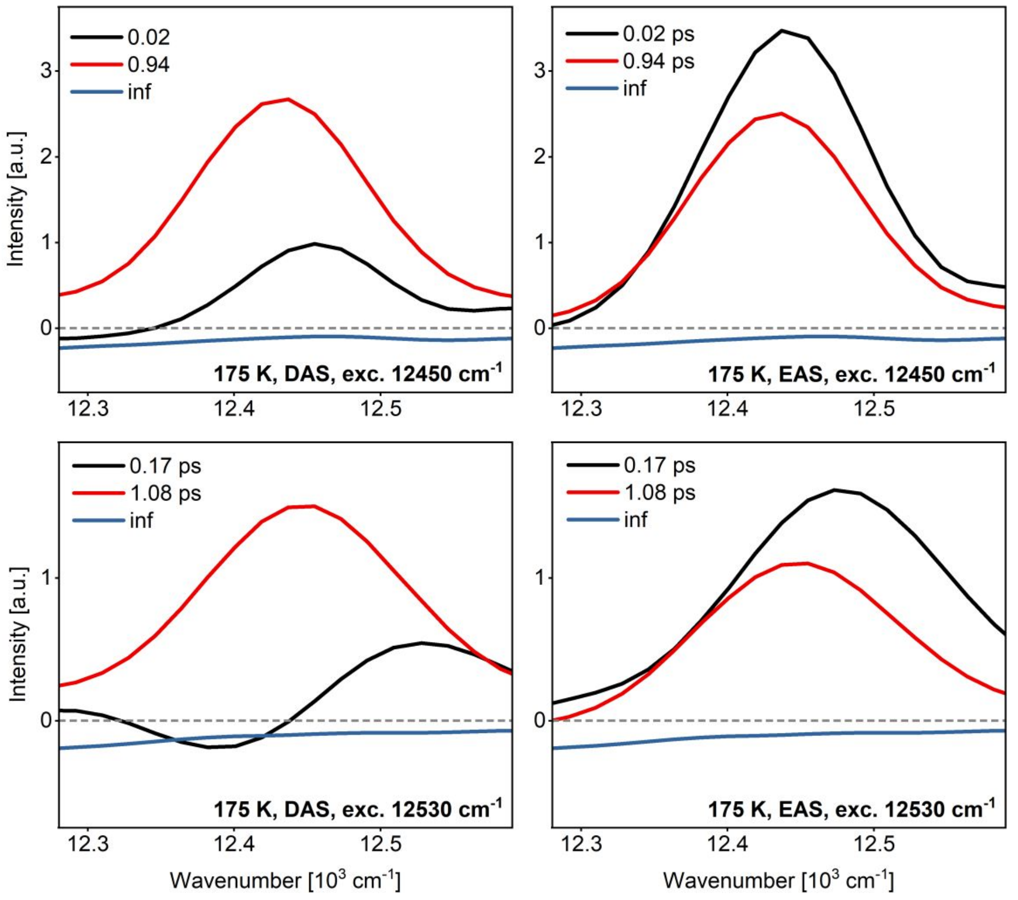
0.02
0.94
inf
0.02 ps
0.94 ps
175 K, DAS, exc. 12450 cm-1
175 K, EAS, exc. 12450 cm-1
0.17 ps
1.08 ps
inf
175 K, DAS, exc. 12530 cm-1
175 K, EAS, exc. 12530 cm-1
Intensity [a.u.]
Wavenumber [10³ cm-1]

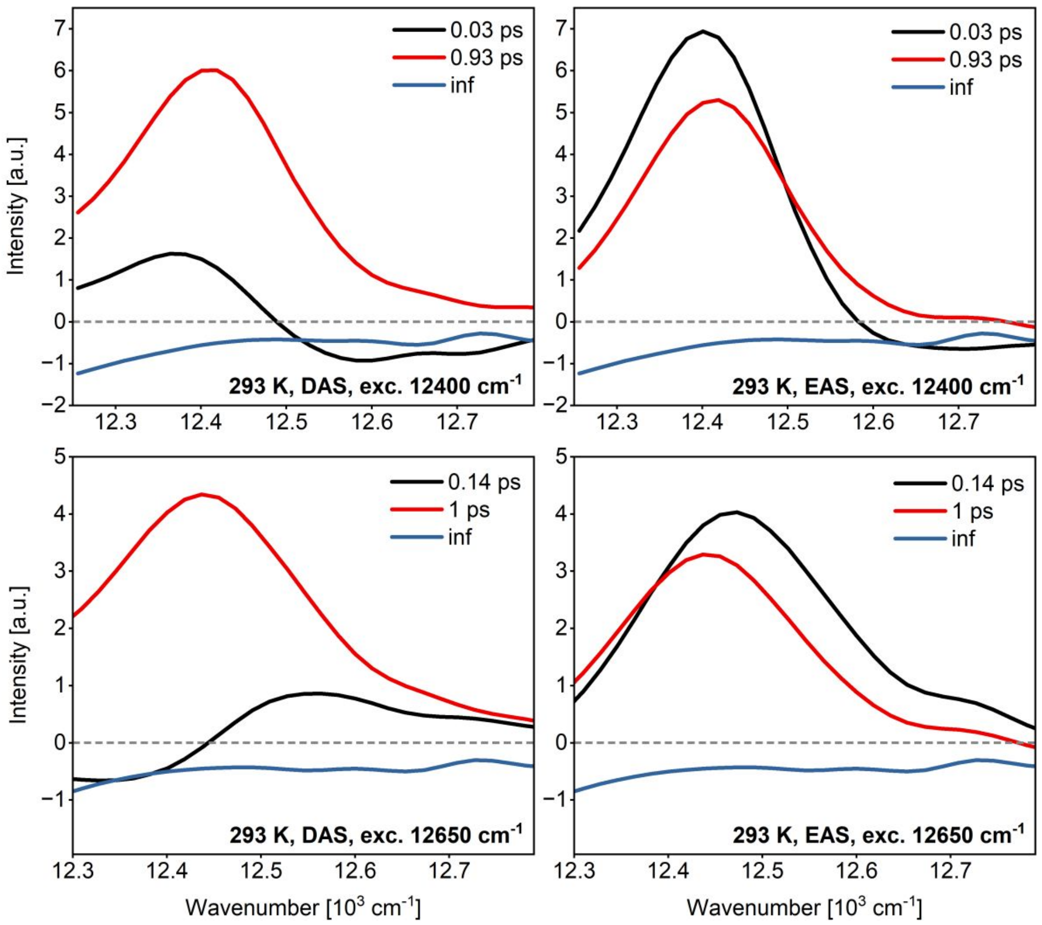

Figure S3

Kinetic analysis (Evolution- and Decay-associated spectra, EAS and DAS, as obtained by global analysis) of the B800 band at different temperatures.

The intraband dynamics of the B800 band in *Rps. acidophila* at 80 K have been described in detail before. Three components are generally needed to fit the data satisfactorily. We refer to previous publications for their detailed interpretation [1,2].

In the sub-ps range, one component displays clear bimodal behavior as a function of excitation frequency. The "dispersive" DAS lineshape appearing after blue-edge excitation demonstrates a clear association with downhill energy transfer. The small amplitude and less well-defined DAS lineshape appearing after red-edge excitation, on the other hand, suggests that, under these conditions, this component is related to inertial solvation or other electronic relaxation processes in the immediate environment rather than energy transfer. The bimodal behavior observed for this component can thus be interpreted as a result of the interplay of different pathways of energy dissipation. The lifetime of this component (partly) varies with temperature: after blue-edge excitation, it approaches 400-500 fs (200 fs) at 80 K (293 K). However, relaxation after red-edge excitation remains roughly the same (~50-100 fs) across temperatures.

The subsequent dynamics are dominated by B800→B850 energy transfer, leading to a complete loss of the B800 signal in 1-2 ps. The exact transfer time decreases with increasing temperature.

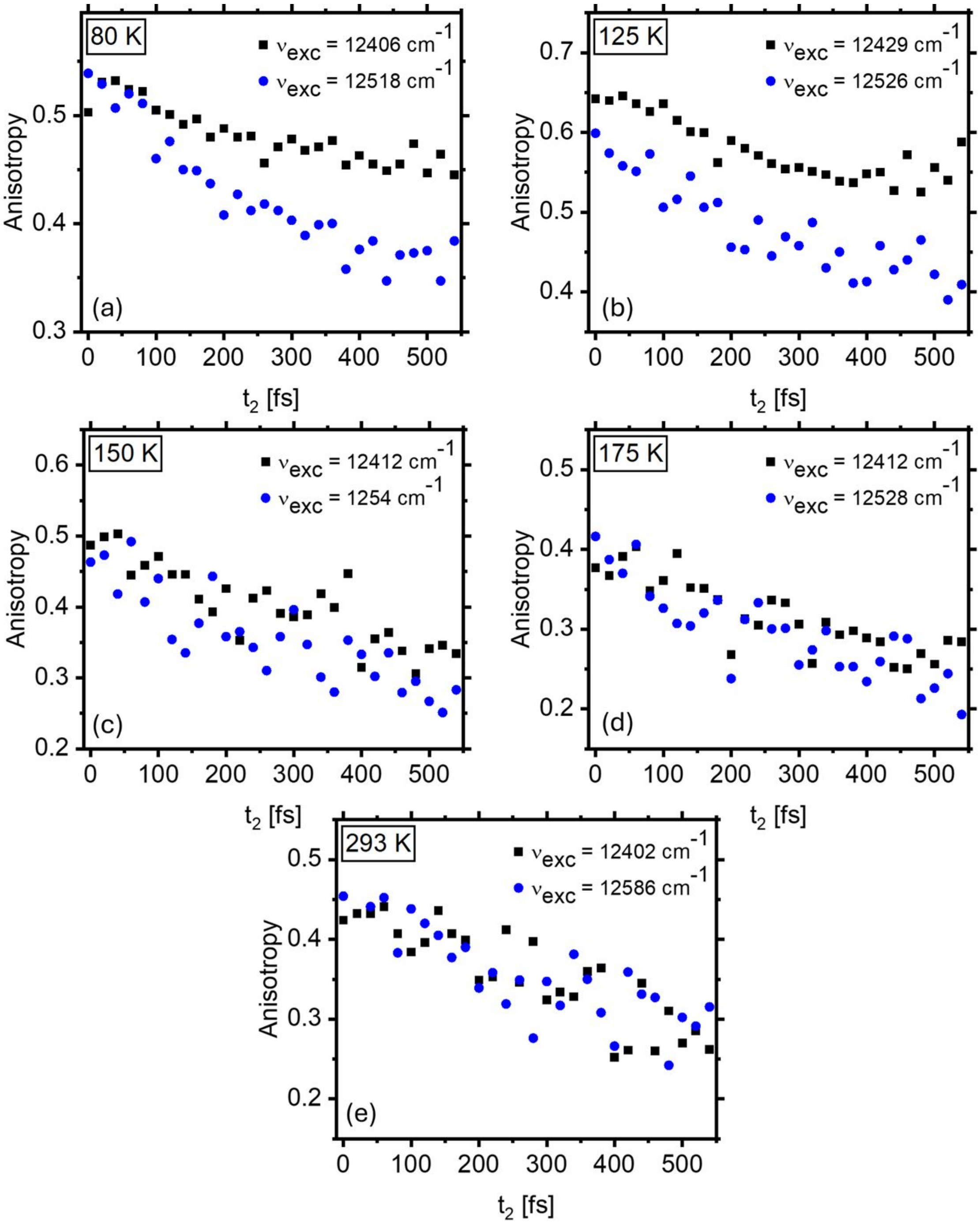

Figure S4

Selected anisotropy traces of the B800 band after excitation at the red- and blue edge at different temperatures. The probe wavelength was chosen to be the respective peak maximum. Time-resolved anisotropy supplies information on the mobility of the excitations in B800. At 80/125 K and after blue-edge excitation, the anisotropy decays rapidly, implying limited but non-negligible exciton mobility. After red-edge excitation, however, the depolarization is slow compared to the B800 lifetime. This is expected for motion between weakly coupled pigments with substantial static disorder. At and above 150 K, the depolarization rate is the same regardless of the excitation energy. This could be because, in the latter regime, the thermal energy is comparable to the width of the B800 band (σ(B800) = 150 cm^{-1}; $k_BT(150K)/hc$ = 104 cm^{-1}, $k_BT(175K)/hc$ = 122 cm^{-1}). As a result, all states can be populated at any time, regardless of excitation energy.

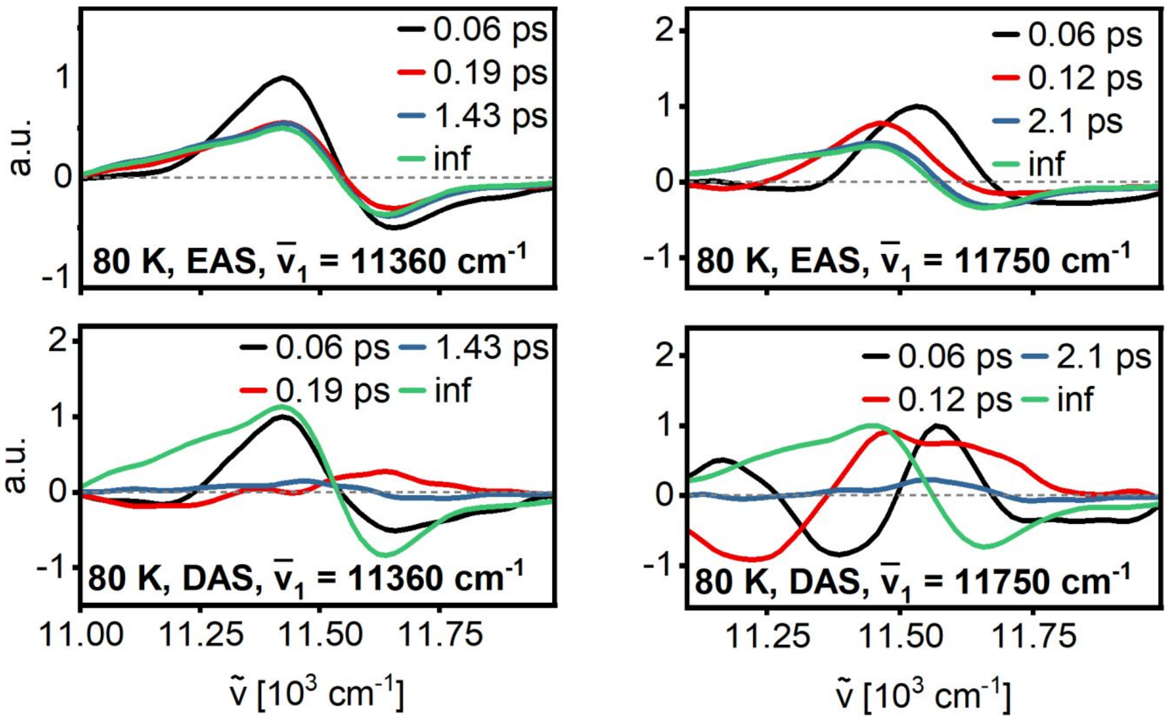
80 K, EAS, $\bar{v}_1$ = 11360 cm^{-1}
0.06 ps
0.19 ps
1.43 ps
inf
80 K, DAS, $\bar{v}_1$ = 11360 cm^{-1}
80 K, EAS, $\bar{v}_1$ = 11750 cm^{-1}
0.06 ps
0.12 ps
2.1 ps
inf
80 K, DAS, $\bar{v}_1$ = 11750 cm^{-1}
a.u.
$\tilde{v}$ [10^3 cm^{-1}]

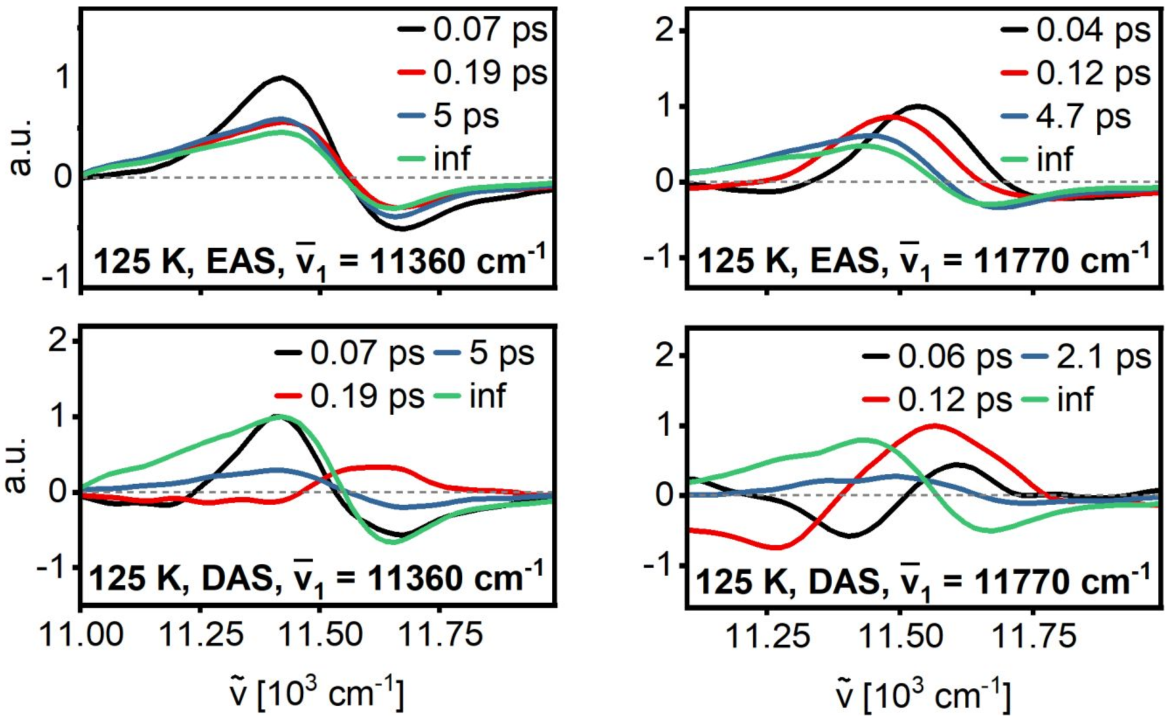
125 K, EAS, $\bar{v}_1$ = 11360 cm^{-1}
0.07 ps
0.19 ps
5 ps
inf
125 K, DAS, $\bar{v}_1$ = 11360 cm^{-1}
125 K, EAS, $\bar{v}_1$ = 11770 cm^{-1}
0.04 ps
0.12 ps
4.7 ps
inf
125 K, DAS, $\bar{v}_1$ = 11770 cm^{-1}
0.06 ps
2.1 ps
a.u.
$\tilde{v}$ [10^3 cm^{-1}]

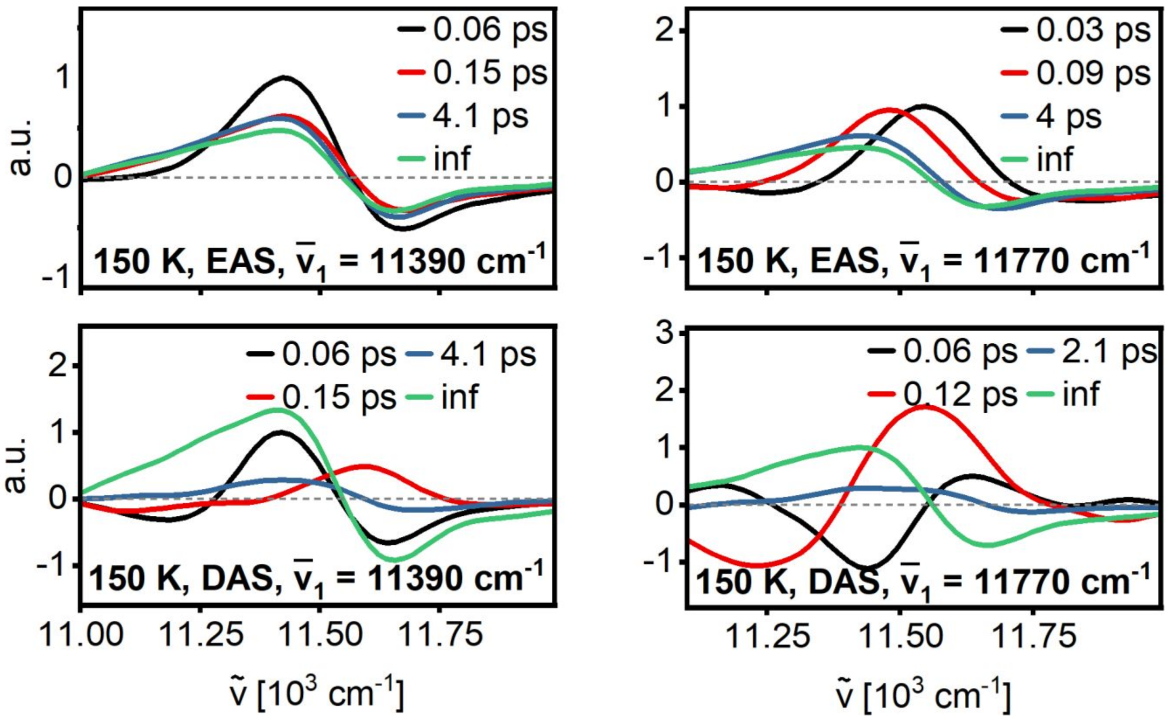
150 K, EAS, $\bar{v}_1$ = 11390 cm^{-1}
0.06 ps
0.15 ps
4.1 ps
inf
150 K, DAS, $\bar{v}_1$ = 11390 cm^{-1}
0.06 ps
4.1 ps
0.15 ps
inf
150 K, EAS, $\bar{v}_1$ = 11770 cm^{-1}
0.03 ps
0.09 ps
4 ps
inf
150 K, DAS, $\bar{v}_1$ = 11770 cm^{-1}
0.06 ps
2.1 ps
0.12 ps
inf
a.u.
$\tilde{v}$ [10^3 cm^{-1}]

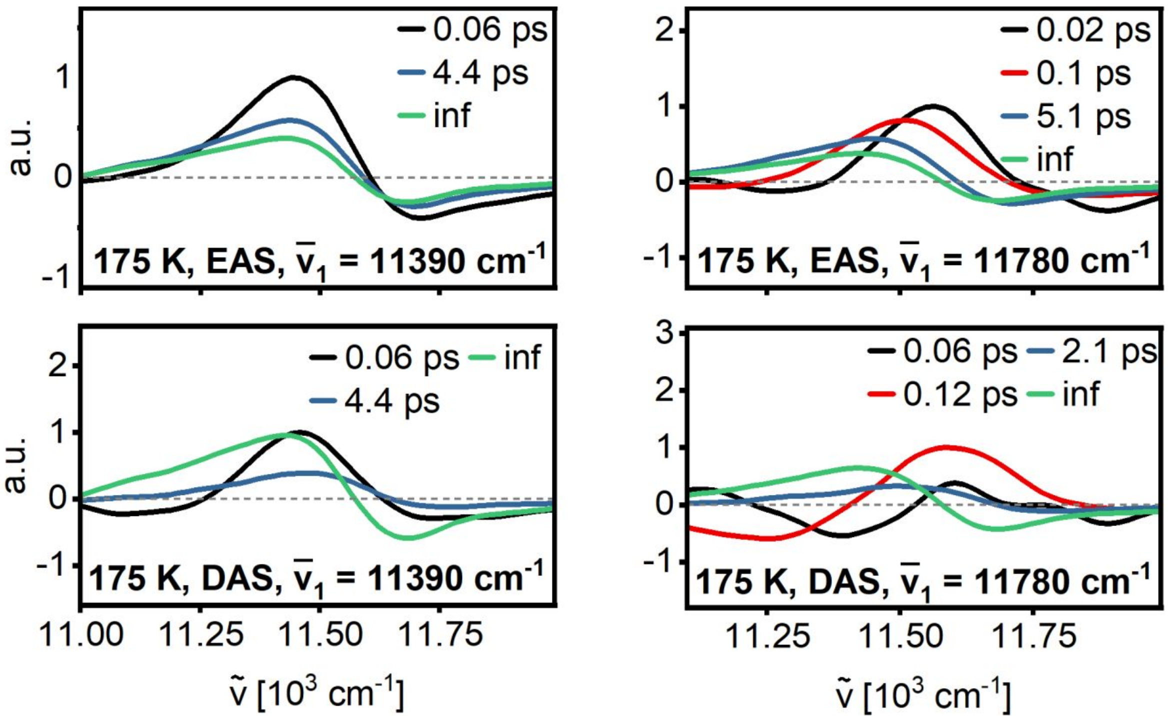
175 K, EAS, $\bar{v}_1$ = 11390 cm^{-1}
0.06 ps
4.4 ps
inf
175 K, DAS, $\bar{v}_1$ = 11390 cm^{-1}
0.06 ps
inf
4.4 ps
175 K, EAS, $\bar{v}_1$ = 11780 cm^{-1}
0.02 ps
0.1 ps
5.1 ps
inf
175 K, DAS, $\bar{v}_1$ = 11780 cm^{-1}
0.06 ps
2.1 ps
0.12 ps
inf
a.u.
$\tilde{v}$ [10^3 cm^{-1}]

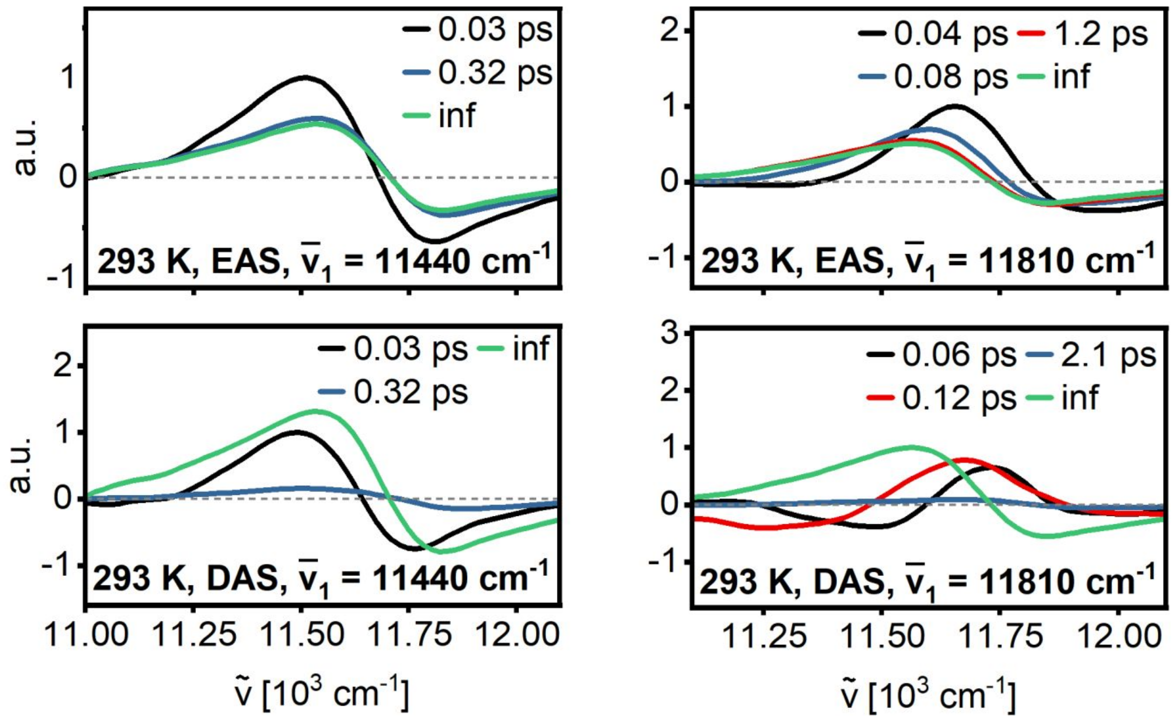

Figure S5

Kinetic analysis (Evolution- and Decay-associated spectra, EAS and DAS, as obtained by global analysis) of the B850 band at different temperatures. A detailed analysis of each kinetic component can be found in the main text.

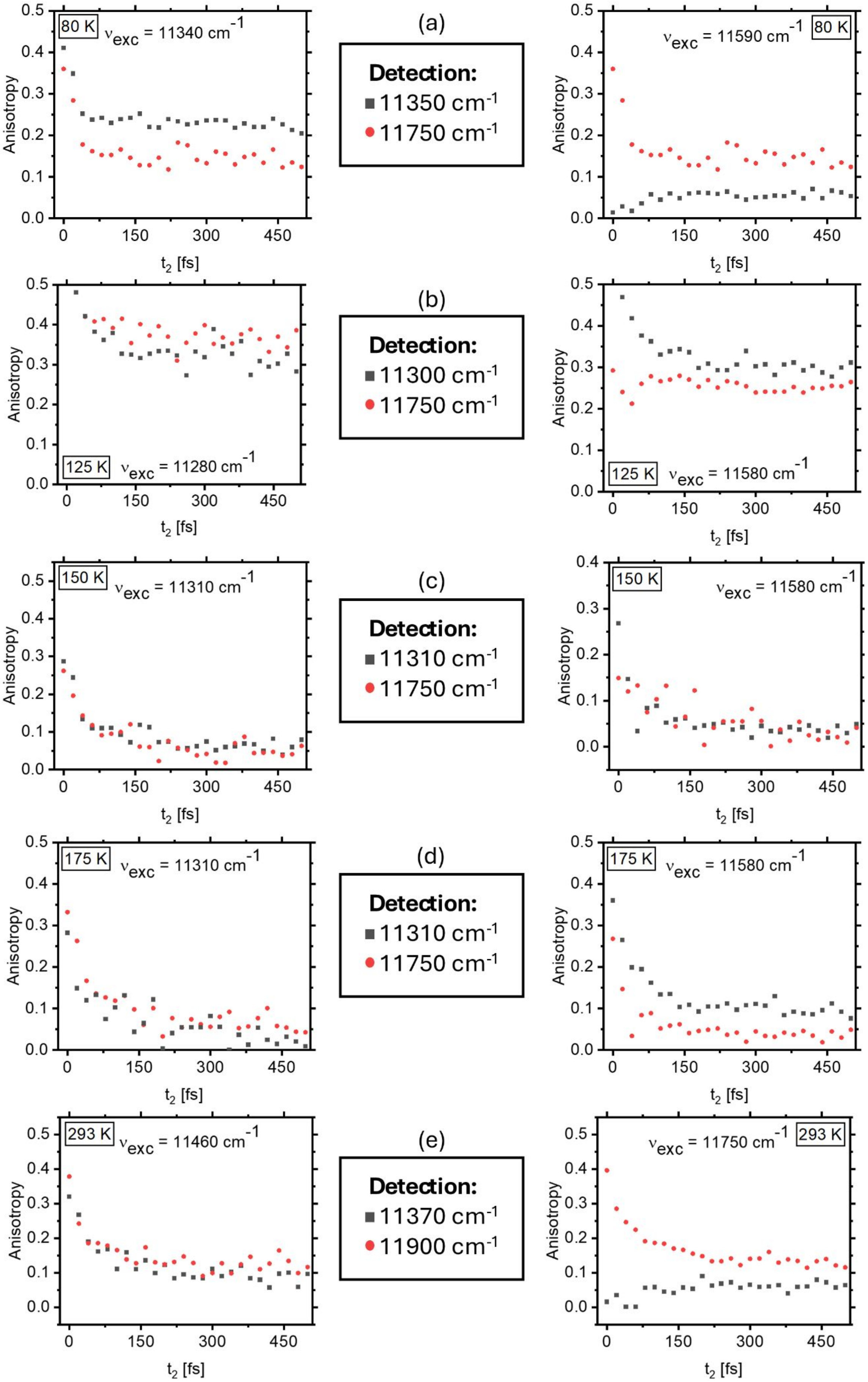
(a)
Detection:
11350 cm-1
11750 cm-1
80 K
νexc = 11340 cm-1
νexc = 11590 cm-1
(b)
Detection:
11300 cm-1
11750 cm-1
125 K
νexc = 11280 cm-1
νexc = 11580 cm-1
(c)
Detection:
11310 cm-1
11750 cm-1
150 K
νexc = 11310 cm-1
νexc = 11580 cm-1
(d)
Detection:
11310 cm-1
11750 cm-1
175 K
νexc = 11310 cm-1
νexc = 11580 cm-1
(e)
Detection:
11370 cm-1
11900 cm-1
293 K
νexc = 11460 cm-1
νexc = 11750 cm-1
Anisotropy
t2 [fs]

Figure S6
Single-point anisotropy traces of the B850 band after excitation at the red- and blue edge of the band at different temperatures. For each excitation condition, the anisotropy was probed at the red edge of the GSB/SE feature and on the blue side of the ESA feature.
All traces show an initial fast decay happening in about 50 fs. This component might be related to the initial dephasing dynamics, but its value is also close to that of the excitation pulse cross-correlation, so a definitive assignment is not possible.
At temperatures of 150 K and above, we observe fast depolarization to r=0.1 regardless of excitation and probing frequency. However, consistent with previous reports, we find that at 80-125 K, no depolarization occurs after exciting and probing at the red edge of the band, indicating that energy relaxation under these conditions involves on average less than one spatial transfer step. Results from global analysis (cf. main text) indicate that, even in this spectral range, an ultrafast (<200 fs) relaxation process occurs, suggesting the presence of a local relaxation process happening from, for example, a shallow trap state ("self-trapping").
At or slightly above 150 K, the thermal energy is already enough to overcome the trap depth regardless of excitation energy. The depth of such a trap state could then be estimated to be at or above $k_BT(150K) = 104\ cm^{-1}$ and below $k_BT(175K) = 122\ cm^{-1}$.
We note that the absolute values of the anisotropy at 80K are slightly lower than reported in our earlier work [1,2] – likely due to polarizer misalignment. This discrepancy does not affect any conclusions made in this work.

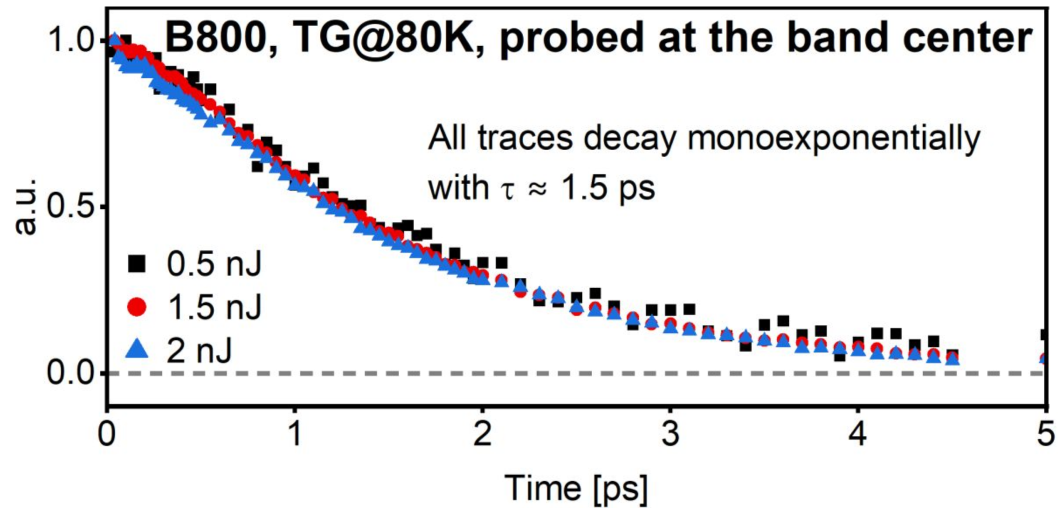

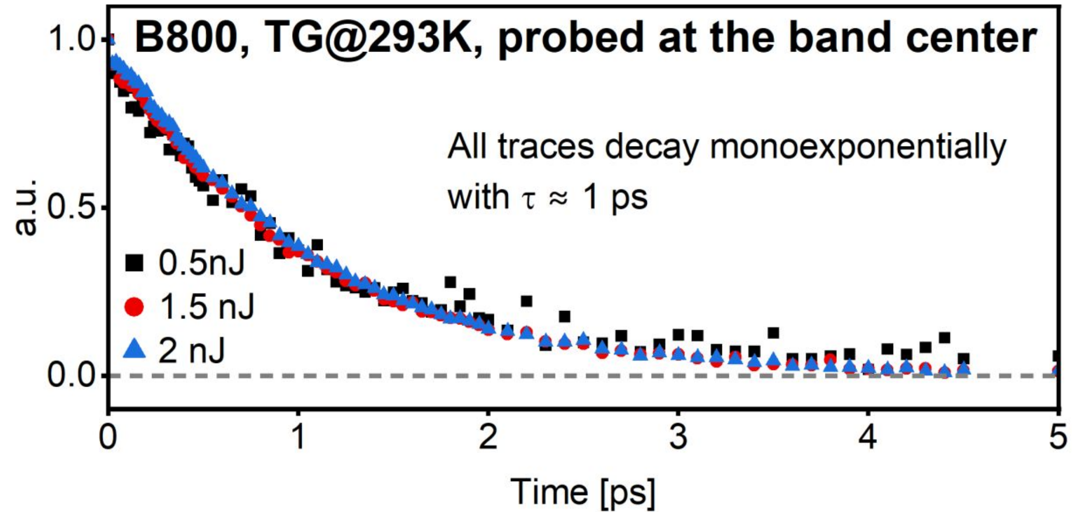

Figure S7

Cuts through the transient grating data along Δt showing the kinetics of the B800 band as a function of pump power at 80 K and room temperature. All traces probe the center of the B800 band.

In LH2 under our measurement conditions, the source of nonlinearity in the transient response is the exciton-exciton annihilation. In the B800 band, the excitons move slowly both around the ring and in their transfer to the B850 ring. Regardless of pump power, we always observe the same monoexponential decay with τ = 1 ps at room temperature and τ = 1.5 ps at 80 K. This time constant corresponds to B800→B850 energy transfer. Since there is no evidence for a distortion or shortening of the kinetics at higher pump powers, we conclude that exciton-exciton annihilation does not play a role in the B800 band under our experimental conditions.

As the transient grating signal of the B800 band within the first couple of hundreds of fs can be considered annihilation-free, it reflects the single-excitation dynamics, and scales accordingly with the pulse intensity. We make use of this scaling factor to correct for experimental attenuation of the signal on the detector, carried out to maximize signal to noise in the measurement, and thus isolate the B850 nonlinear signals.

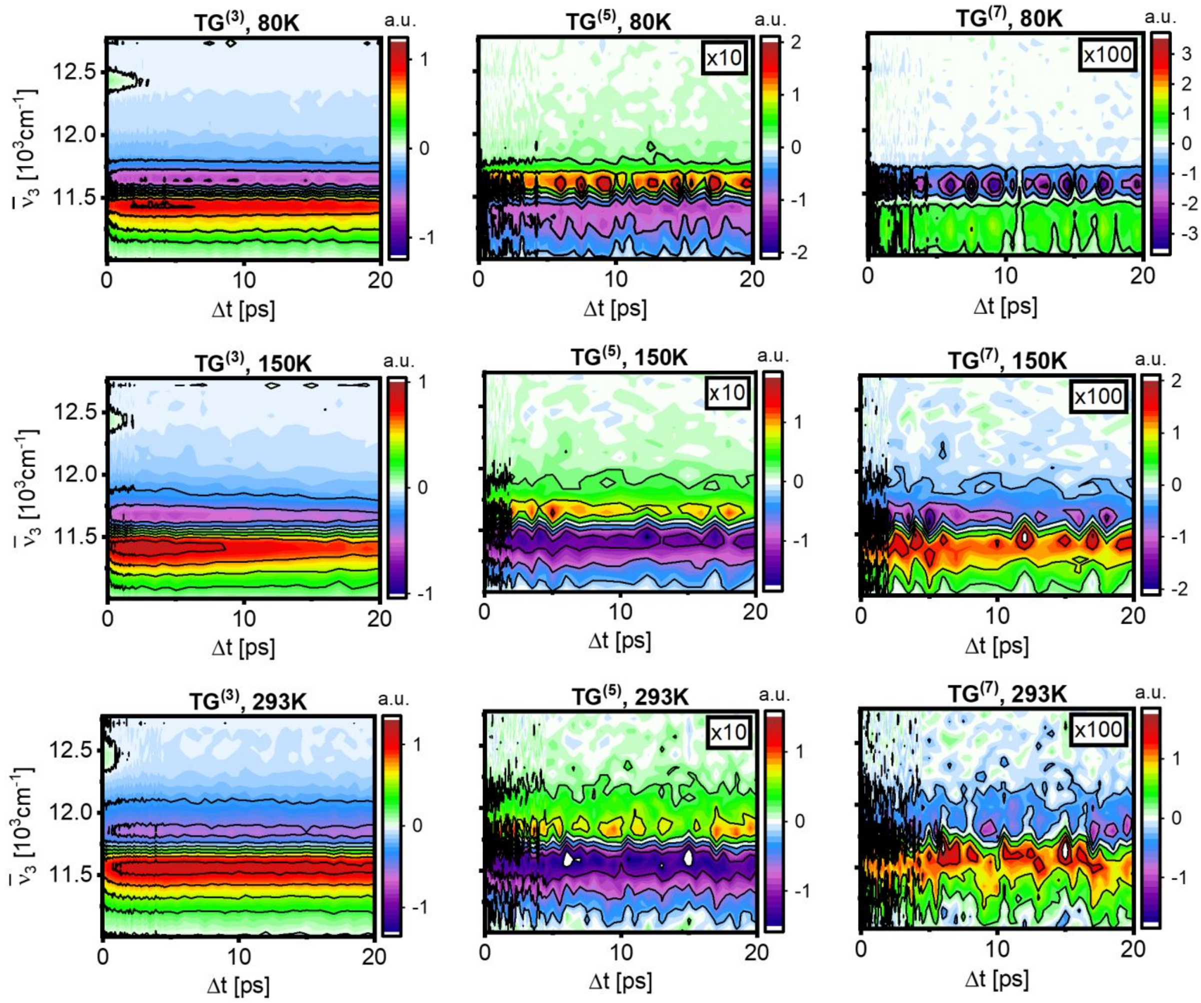

Figure S8

TG$^{(3)}$, TG$^{(5)}$ and TG$^{(7)}$ signals extracted from the intensity-dependent TG measurements. We show representative data at 80 K, 150 K and 293 K, but the analysis was performed for experiments at 125 K and 175 K as well. As expected, the signal strength decreases rapidly with signal order.

Figure S9

Global analysis of the (decomposed) transient grating datasets corresponding to the third- and fifth order signals at different temperatures. For ease of comparison, the $TG^{(3)}$ data is plotted following the sign convention for 2DES (positive GSB and SE, negative ESA in third order). The $TG^{(5)}$ data is plotted with the opposite sign according to the relationship between third- and fifth-order data.

Fitting the third order TG dataset requires three kinetic components. The fastest one (<100 fs) is likely associated to dephasing. The second component represents mainly the B800→B850 transfer. Compared to the lifetimes obtained for B800→B850 transfer from 2DES, the TG3 kinetic components are consistently shorter. This is likely due to intraband relaxation being mixed into this component, as we cannot resolve it individually. The third (very long) component is assigned to ground state recovery.

The fifth order datasets also require three components to obtain a satisfactory fit. As done for the third order data, there is a very long component that we assign to ground state recovery. An intermediate component represents B800→B850 transfer and was kept fixed in the fits using the values obtained from the 2DES data. Finally, we see an ultrafast component that is heavily temperature dependent and varies between 450 fs (80 K) and 50 fs (293 K). We assign this component to exciton-exciton annihilation.

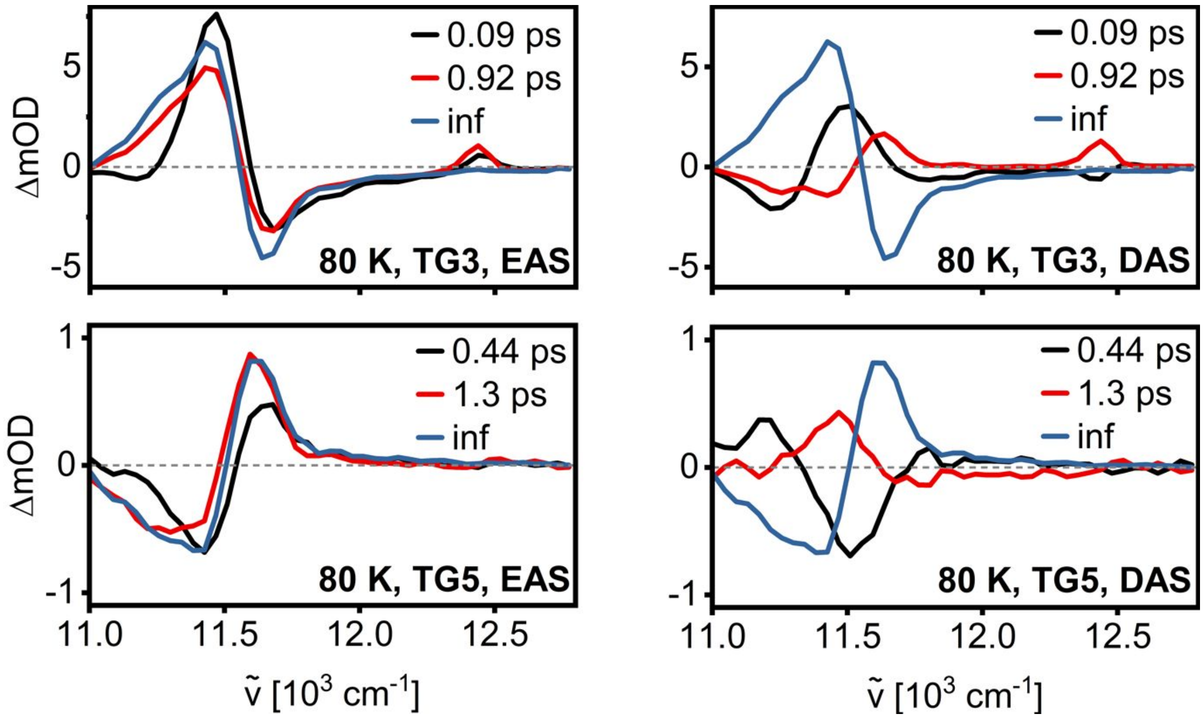

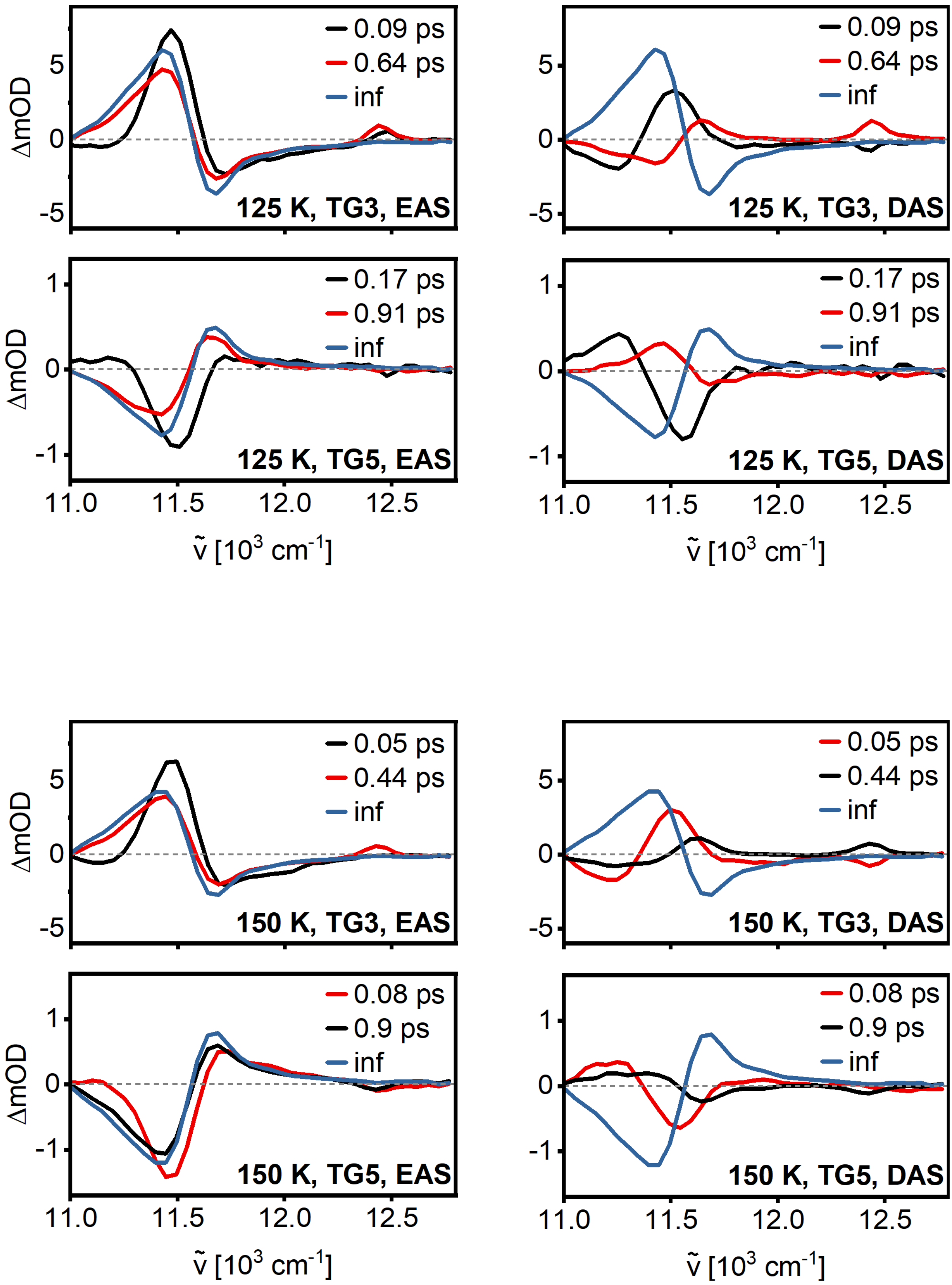

0.09 ps
0.64 ps
inf
125 K, TG3, EAS
0.09 ps
0.64 ps
inf
125 K, TG3, DAS
0.17 ps
0.91 ps
inf
125 K, TG5, EAS
0.17 ps
0.91 ps
inf
125 K, TG5, DAS
ΔmOD
ṽ [10³ cm⁻¹]
0.05 ps
0.44 ps
inf
150 K, TG3, EAS
0.05 ps
0.44 ps
inf
150 K, TG3, DAS
0.08 ps
0.9 ps
inf
150 K, TG5, EAS
0.08 ps
0.9 ps
inf
150 K, TG5, DAS

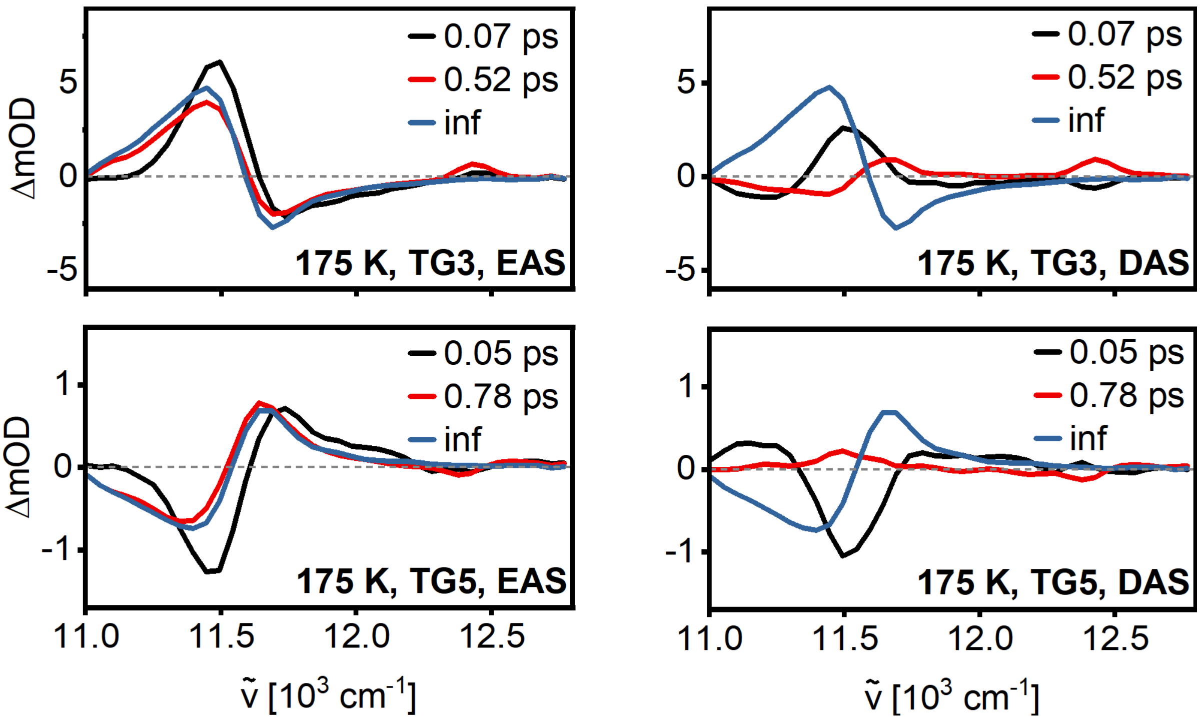
0.07 ps
0.52 ps
inf
175 K, TG3, EAS
0.07 ps
0.52 ps
inf
175 K, TG3, DAS
0.05 ps
0.78 ps
inf
175 K, TG5, EAS
0.05 ps
0.78 ps
inf
175 K, TG5, DAS
ΔmOD
ṽ [10³ cm⁻¹]

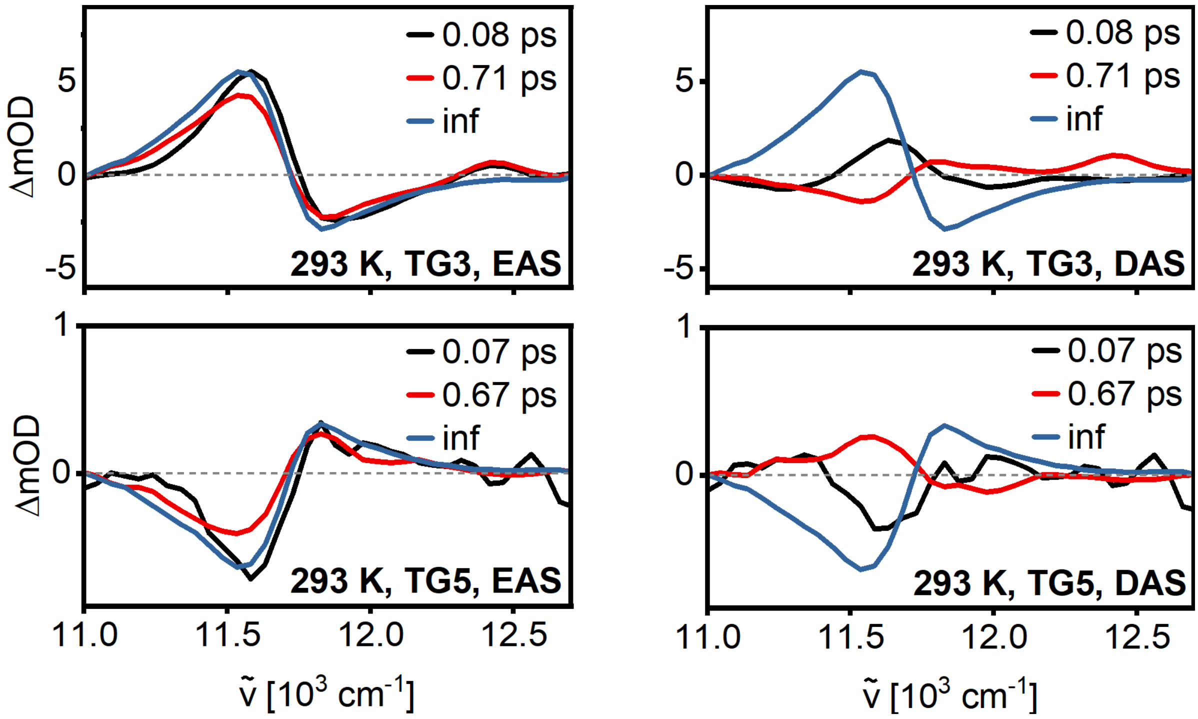
0.08 ps
0.71 ps
inf
293 K, TG3, EAS
0.08 ps
0.71 ps
inf
293 K, TG3, DAS
0.07 ps
0.67 ps
inf
293 K, TG5, EAS
0.07 ps
0.67 ps
inf
293 K, TG5, DAS
ΔmOD
ṽ [10³ cm⁻¹]

Supporting Section 1: Experimental Details

1. Technical details

The instrument used for 2DES experiments is described in detail elsewhere [3]. In summary, a 1027 nm Yb:KGW laser (Pharos, Light Conversion ltd.) was used to pump a lab-built noncollinear optical parametric amplifier, the pulses of which were compressed by a combination of chirped mirrors and a fused silica prism compressor. The output was pulses of ~12 fs in duration with a spectrum centered at 830 nm. The beams were focused into a ~160 µm diameter spot in the sample. All experiments were done at a 20 kHz repetition rates, and for temperatures T = 80 K, 125 K, 150 K, 175 K and 293 K. Cryogenic measurements were performed in a liquid nitrogen flow cryostat (Oxford Instruments). For 2DES measurements, the coherence time was scanned from -230 to 550 fs with 2 fs steps, resulting in a spectral resolution of 33 cm^{-1} on the $\tilde{\nu}_1$ axis; the resolution on the $\tilde{\nu}_3$ axis was 55 cm^{-1}. The linear polarizations of the pulses were independently controlled by a combination of a broadband quarter-wave plate and linear wire-grid polarizers in each beam. To avoid depolarization effects, we measured population dynamics at the magic angle condition (<54.7, 54.7, 0, 0>, given numbers in degrees), while the anisotropy was calculated as usual from the combination of the <0, 0, 0, 0> and <90, 90, 0, 0> sequences of linearly polarized pulses. To avoid dynamics related to exciton-exciton annihilation, the pulse energy was kept at 0.6 nJ (ca. $8\cdot10^{12}$ photons/cm^2). The TG signal was collected under magic angle conditions with the same setup by setting the coherence time to 0. For each temperature, we collected TG data at pump energies per pulse of 0.5 nJ, 1.5 nJ and 2 nJ (ca. $8.2\cdot10^{12}$, $2.4\cdot10^{13}$, $3.3\cdot10^{13}$ photons/cm^2 assuming a central wavelength of 830 nm).

2. Sample preparation and experiment

LH2 from *Rps. acidophila* was extracted, isolated, and purified according to earlier detailed procedures [4]. The purified samples were stored at -20 °C in a buffer until immediately before use. Before the experiment, the LH2 stock solution was diluted in TRIS-HCl buffer containing LDAO, glucose, glucose oxidase, and catalase. The resulting buffer solution was mixed to a 1:2 ratio with glycerol to reach an absorbance of ~0.4 in a 200 µm optical path cell, whereafter the sample was immediately frozen and measured.

3. Kinetic Modeling, data analysis and visualization

The global analysis was done using the open-source software Glotaran [5]. The numerical modeling was done in MATLAB.

Supporting Section 2: Modeling

We perform a simplified Monte Carlo simulation in which excitons move along the ring in a discrete random walk. We take the B850 ring to consist of nine effective sites. The hopping rate between sites (k_{jump}) is a constant, as we do not consider thermally activated transfer. If the latter did play a role, the expression for k_{jump} could be trivially modified to $k_{jump} = Ae^{-E_{jump}/k_B T}$, where E_{jump} is the energy barrier for uphill transfer and A is a preexponential factor describing the frequency of attempted crossings.
At each site, excitons can get trapped with a rate k_{trap}. The trap depth is set to E_{trap}. Once trapped, the detrapping rate is given by $k_{detrap} = k_{trap} \cdot e^{-E_{trap}/k_B T}$ according to detailed balance. When two excitons meet at the same site, they annihilate with the probability p_{anni}.

We set our parameters as follows:

The depth of the traps is set to E_{trap} = 120 cm^{-1} as obtained from the analysis of the 2DES data. Site-to-site hopping in LH2 has been reported to occur in about 80 fs. Since hopping can occur to the left or right neighbor, the residence time on a single site is half the hopping time, or 40 fs [6]. We thus set k_{jump} and k_{trap} to the same value of 1/40 = 0.025 fs^{-1}.
Finally, we assume that p_{anni} = 1, meaning that when two excitons meet, they always annihilate. We also assume that the traps are non-annihilating and that EEA only happens on the ring.
For every calculation run, we produce histograms of the time it takes for one exciton to be quenched or to annihilate at each temperature and calculate the mean. We then fit the histogram to an exponential decay (rejecting the first bin representing immediate quenching), thus obtaining an estimate for the rates. The natural logarithm values of the fitted rates are then plotted against 1/T and fitted to a straight line in an Arrhenius plot. The slope of the line multiplied by the Boltzmann constant k_B yields the activation energy for the EEA process.

We point out that our results in this section are qualitative, as our model does not accurately capture the complexity of energy relaxation in LH2. In particular; assuming that each exciton is delocalized over two BChls and that the size does not change with temperature is not realistic. A probability of 1 to annihilate upon encounter may be an overestimation. However, changing the number of sites or reducing the annihilation probability does not change the outcome qualitatively.

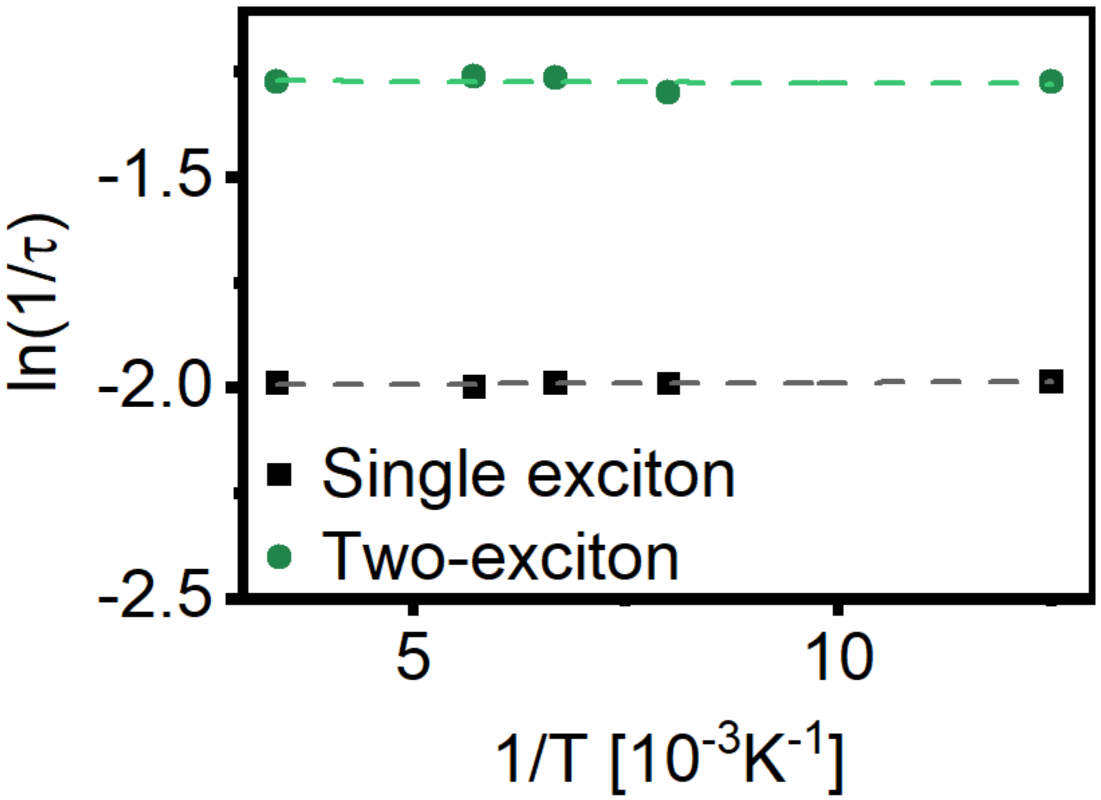

Figure S10
Activation energies for quenching and annihilation when trapping is not considered in the model. Results are shown for a single excitation in the ring with a fixed quenching site and two excitations that can move simultaneously on the ring and annihilate upon meeting. τ is the mean lifetime of the single exciton (black) or of the exciton pair (green). When thermally activated transport is not included, E_a is always zero.

p_{anni}(free)	p_{anni}(2trapped)	p_{anni}(mix)	E_{trap} [cm^{-1}]	k_{trap} [fs^{-1}]	k_{jump} [fs^{-1}]	E_a [cm^{-1}]
1	1	1	120	0.025	0.025	100
1	1	0	120	0.025	0.025	120
1	0	0	120	0.025	0.025	180
1	1	1	240	0.025	0.025	230
1	1	0	240	0.025	0.025	240
1	0	0	240	0.025	0.025	470
1	1	1	60	0.025	0.025	40
1	1	0	60	0.025	0.025	50
1	0	0	60	0.025	0.025	70
1	1	1	120	0.05	0.025	95
1	1	0	120	0.05	0.025	110
1	0	0	120	0.05	0.025	170
1	1	1	120	0.00125	0.025	100
1	1	0	120	0.00125	0.025	170
1	0	0	120	0.00125	0.025	150-200
1	1	0.1	120	0.025	0.025	100
1	1	0.01	120	0.025	0.025	110
1	1	10^{-8}	120	0.025	0.025	120
0.1	0	0	120	0.025	0.025	180
0.01	0	0	120	0.025	0.025	180-190

Table T1

Retrieved activation energies upon variation of the modeling parameters. Here, p_{anni}(free) is the probability of two mobile excitons to annihilate; p_{anni}(2trapped) is the probability of two trapped excitons to annihilate; p_{anni}(mix) is the probability of one mobile and one trapped exciton to annihilate; E_{trap} is the trap depth; k_{trap} is the trapping rate; k_{jump} is the jumping rate; and E_a is the effective retrieved transport barrier.

To explore the modeling parameters, we formally allow two excitons to fall into the same trap. A probability of p_{anni}(2trapped) = 0 for two excitons who can fall into the same trap means that the traps are non-annihilating and is effectively the same as forbidding the simultaneous occupation of one trap by two excitons.

References

(1) Keil, E.; Lokstein, H.; Cogdell, R.; Hauer, J.; Zigmantas, D.; Thyrhaug, E. Light Harvesting in Purple Bacteria Does Not Rely on Resonance Fine-Tuning in Peripheral Antenna Complexes. *Photosynth. Res.* **2024**, *161* (3), 191–201.

(2) Thyrhaug, E.; Schröter, M.; Bukartė, E.; Kühn, O.; Cogdell, R.; Hauer, J.; Zigmantas, D. Intraband Dynamics and Exciton Trapping in the LH2 Complex of Rhodopseudomonas Acidophila. *Journal of Chemical Physics* **2021**, *154* (4).

(3) Augulis, R.; Zigmantas, D.; Hybl, J. D.; Albrecht, A. W.; G Faeder, S. M.; Jonas, D. M. Two-Dimensional Electronic Spectroscopy with Double Modulation Lock-in Detection: Enhancement of Sensitivity and Noise Resistance References and Links. *Optics Express* **2011**, *19* (14).

(4) Gardiner, A. T.; Niedzwiedzki, D. M.; Cogdell, R. J. Adaptation of Rhodopseudomonas Acidophila Strain 7050 to Growth at Different Light Intensities: What Are the Benefits to Changing the Type of LH2? *Faraday Discuss.* **2018**, *207*, 471–489.

(5) Snellenburg, J. J.; Laptenok, S.; Seger, R.; Mullen, K. M.; van Stokkum, I. H. M. Glotaran: A Java-Based Graphical User Interface for the R Package TIMP. *J. Stat. Softw.* **2012,** *49* (3).

(6) Bradforth, S. E.; Jimenez, R.; Van Mourik F., Van Grondelle, R.; Fleming, G. R., Excitation Transfer in the Core Light-Harvesting Complex (LH-1) of Rhodobacter Sphaeroides: An Ultrafast Fluorescence Depolarization and Annihilation Study. *J. Phys. Chem.* **1995,** *99*, 16179-16191.
